# Restoring microglial and astroglial homeostasis using DNA immunization in a Down Syndrome mouse model

**DOI:** 10.1016/j.bbi.2018.10.004

**Published:** 2018-10-25

**Authors:** Tomer Illouz, Ravit Madar, Arya Biragyn, Eitan Okun

**Affiliations:** a The Leslie and Susan Gonda Multidisciplinary Brain Research Center, Bar-Ilan University, Ramat Gan 5290002, Israel; b The Mina and Everard Goodman Faculty of Life Sciences, Bar-Ilan University, Ramat Gan 5290002, Israel; c The Paul Feder Laboratory on Alzheimer’s Disease Research, Bar-Ilan University, Ramat Gan 5290002, Israel; d Laboratory of Molecular Biology and Immunology, NIA, NIH, MD 21224, USA

**Keywords:** Down Syndrome, Ts65Dn, Alzheimer’s disease, Amyloid-β, Vaccine, Microglia, Astrocytes

## Abstract

Down Syndrome (DS), the most common cause of genetic intellectual disability, is characterized by over-expression of the APP and *DYRK1A* genes, located on the triplicated chromosome 21. This chromosomal abnormality leads to a cognitive decline mediated by Amyloid-β (Aβ) overproduction and tau hyper-phosphorylation as early as the age of 40.

In this study, we used the Ts65Dn mouse model of DS to evaluate the beneficial effect of a DNA vaccination against the Aβ_1–11_ fragment, in ameliorating Aβ-related neuropathology and rescue of cognitive and behavioral abilities. Anti-Aβ_1–11_ vaccination induced antibody production and facilitated clearance of soluble oligomers and small extracellular inclusions of Aβ from the hippocampus and cortex of Ts65Dn mice. This was correlated with reduced neurodegeneration and restoration of the homeostatic phenotype of microglial and astroglial cells. Vaccinated Ts65Dn mice performed better in spatial-learning tasks, exhibited reduced motor hyperactivity typical for this strain, and restored short-term memory abilities.

Our findings support the hypothesis that DS individuals may benefit from active immunotherapy against Aβ from a young age by slowing the progression of dementia.

## 1. Introduction

Down Syndrome (DS) or trisomy 21, is the most common chromosomal abnormality found in humans and the most prevalent genetic cause of intellectual disability, affecting 1 in 850–1000 infants (Lejeune et al., 1959; Shin et al., 2009). The Amyloid precursor protein (*APP*) and dual specificity tyrosine-phosphorylation-regulated kinase 1A (*DYRK1A*) genes, located within the triplicated human chromosome 21 (Hsa21) (Korenberg et al., 1990; Rueda et al., 2012) are overexpressed in DS individuals. This results in Alzheimer’s disease (AD)-like neuropathology that can be found as early as 17-years of age (Burger and Vogel, 1973) and in the vast majority of patients over 40 (Mann, 1988). AD is cerebrally manifested by accumulation of extracellular deposits of Amyloid-β (Aβ) in form of plaques (Goedert, 2015), neuritic plaques (Mattson, 2004), elevated levels of neurotoxic Aβ oligomers (Benilova et al., 2012), cerebral amyloidic angiopathy (Ghiso et al., 2010) and intraneuronal accumulation of hyperphosphorylated tau protein in from of tangles (Goedert, 2015). These neuropathological features promote neuronal loss, brain atrophy and severe cognitive impairments (Mattson, 2004). Oligomeric Aβ induces membrane-associated oxidative stress that impairs synaptic plasticity and causes neuritic and tau hyperphosphorylation (Olkhanud et al., 2012). Accumulation of Aβ triggers harmful inflammatory responses in microglia (Walker and Lue, 2015; Yin et al., 2017) and astrocytes (Garwood et al., 2017; Liddelow et al., 2017; Verkhratsky et al., 2010) which in turn promote infiltration of Aβ-specific T cells into the brain (Ferretti et al., 2016). Similarly to heritable forms of AD, DS features elevated levels of soluble Aβ oligomers, accumulation of extracellular Aβ and neuritic plaques, cerebral amyloid angiopathy, intracellular tangles and cerebral inflammation, all promoting neuronal loss, white matter degeneration and cognitive decline (Head et al., 2016). However, distinct genetic background, including life-long overexpression of APP and DYRK1A, distinct DS from sporadic and heritable AD, leading to a unique microglial phenotype in these patients (Wilcock et al., 2015).

The Ts65Dn mouse model of DS encompasses a partial trisomy of mouse chromosome 16 (Mmu 16), which includes 92 genes orthologous to Hsa21 (Davisson et al., 1993). The cognitive and behavioral phenotype of Ts65Dn mice includes delayed motor acquisition, impaired coordination (Costa et al., 1999; Escorihuela et al., 1995), hyperactivity, reduced attention (Coussons-Read and Crnic, 1996; Escorihuela et al., 1995; Holtzman et al., 1996; Reeves et al., 1995) and impaired hippocampal-dependent functions such as contextual fear conditioning, working memory and long-term spatial memory (Belichenko et al., 2007; Demas et al., 1996; Escorihuela et al., 1998; Fernandez and Garner, 2008; Martinez-Cue et al., 2002; Reeves et al., 1995; Sago et al., 2000; Salehi et al., 2009).

The cerebral abnormalities found in Ts65Dn include reduced brain volume (Aldridge et al., 2007; Belichenko et al., 2007; Bianchi et al., 2010b; Chakrabarti et al., 2007; Contestabile et al., 2008; Llorens-Martin et al., 2010; Lorenzi and Reeves, 2006; Martinez-Cue et al., 2002), reduced neuronal density (Bianchi et al., 2010b; Contestabile et al., 2007; Insausti et al., 1998; Kurt et al., 2004; Lorenzi and Reeves, 2006; Rueda et al., 2010), impaired neurogenesis (Bianchi et al., 2010a; Bianchi et al., 2010b; Chakrabarti et al., 2007; Clark et al., 2006; Rueda et al., 2005; Trazzi et al., 2011), reduced density and impaired morphology of dendritic spines and increased synaptic cleft (Belichenko et al., 2009; Belichenko et al., 2007; Belichenko et al., 2004; Dierssen et al., 2003; Popov et al., 2011), increased number of inhibitory synapses and reduced number of excitatory synapses (Belichenko et al., 2004; Kurt et al., 2004; Rueda et al., 2010), increased number of GA-BAergic neurons (Perez-Cremades et al., 2010), and impaired hippocampal long-term potentiation (Kleschevnikov et al., 2004; Siarey et al., 1997). Importantly, although Ts65Dn mice exhibit age-related increase in APP (Seo and Isacson, 2005) and Aβ (Netzer et al., 2010) levels in the cortex and hippocampus, they do not show plaques pathology (Lomoio et al., 2009). Previous reports however, indicate the presence of small amyloidic extracellular inclusions in the deep granular cell layer of the cerebellum of Ts65Dn mice (Lomoio et al., 2009).

Microglial cells play a crucial role in the pathogenesis of AD along with other neurodegenerative conditions. Little is known, however, about the microglial response in DS. Similarly to AD, DS is characterized by morphological and functional alteration in reactive microglia (Xue and Streit, 2011). A recent study, showed a unique microglial phenotype in human DS specimen, distinct from microglia in sporadic forms of AD (Wilcock et al., 2015), manifested by elevated levels of the M1 markers, IL-1β, IL-6, TNFα, the M2a markers, CHI3L1, IL-1Ra, and the M2b markers, CD86, FCGR1.

In addition to microglial cells, astrocytes play a central role in the pathogenesis of AD (Garwood et al., 2017; Guenette, 2003; Liddelow et al., 2017; Rodriguez-Arellano et al., 2016; Verkhratsky et al., 2010) and DS (Chen et al., 2014; Lockrow et al., 2012; Lu et al., 2011; Sebastia et al., 2004). Astrocytes contribute to Aβ clearance and are involved in maintaining tissue homeostasis (Garwood et al., 2017; Guenette, 2003; Liddelow et al., 2017; Sollvander et al., 2016; Wyss-Coray et al., 2003). However, oligomeric Aβ promotes astrocyte-mediated inflammation through the secretion of inflammatory molecules such as IL-1β, nitric oxide synthase (iNOS) and in turn, overproduction of nitric oxide (NO) (White et al., 2005). S100β, expressed by a subtype of mature astrocytes that ensheath blood vessels (Wang and Bordey, 2008), is triplicated in DS (Chen et al., 2014). Previous reports showed that S100β overexpression induces the expression of iNOS and stimulates NO generation by astrocytes (Chen et al., 2014; Hu et al., 1996). Under brain injury and neurodegenerative diseases, reactive microglia promote A1-reactive astrocytes (Liddelow et al., 2017), which in turn can further promote neurodegeneration by secretion of neurotoxins and complement components that drive synapses degeneration. A1 astrocytes also lose their ability to maintain tissue homeostasis by promoting neuronal growth, neuronal survival and synapse formation (Liddelow et al., 2017).

As DS can be diagnosed as early as in utero, and since Aβ burden is a significant contributing factor to the DS neuropathology, individuals with DS can benefit from Aβ-directed immunotherapies at an early age. To test whether an active anti-Aβ immunization (Morgan et al., 2000; Movsesyan et al., 2008; Schenk et al., 1999) can exert a therapeutic effect in DS, we vaccinated Ts65Dn and healthy control mice with Aβ-CoreS, a vaccine shown to be beneficial in the 3xTgAD mouse model of AD (Olkhanud et al., 2012). This vaccine induces the expression of an Aβ_1–11_ peptide fused to the Hepatitis-B surface antigen (HBsAg), a primary component of the Hepatitis-B virus (HBV) vaccine (Zhao et al., 2006). This vaccine also contains the Hepatitis-B capsid antigen (HBcAg) to provide a T helper response that promotes antibody production by B-lymphocytes. Ts65Dn mice and healthy controls were immunized at 6 months of age and were tested for various cognitive behaviors (i.e. spatial memory, short-term memory, exploratory behavior and anxiety threshold, Supp. Table 1). Mice were sacrificed at 15 m of age for biochemical and histological analysis. Vaccinating Ts65Dn mice facilitated clearance of cerebral soluble oligomers and small extracellular inclusions of Aβ and reduced serum and Aβ levels. Furthermore, we found normalization in the levels of hyperphosphorylated tau in the hippocampus of vaccinated mice, along with restoration of the homeostatic phenotype of microglial and astroglial cells. Vaccinated Ts65Dn mice performed better in long-term spatial-learning and memory tasks, exhibited reduced motor hyperactivity typical for this strain, and retained hippocampal-dependent short-term memory capacity.

## 2. Materials and methods

### 2.1. Study design

This study aims at targeting Aβ-related neuropathology and cognitive decline in a mouse model of DS (Reeves et al., 1995). In contrast to sporadic Alzheimer’s disease (AD), DS can be detected as early as in-utero. As the majority of DS individuals will develop AD-like neuropathological features by early adulthood (Burger and Vogel, 1973), we hypothesized that vaccinating DS mice against Aβ at a young age might slow the progression of Aβ-related pathologies. Unlike sporadic AD, AD-related pathology in DS patients exhibits an early onset with rapid progression (Lockrow et al., 2012; Mann, 1988; Nelson et al., 2001). It is therefore essential to develop early-intervention treatments that target Aβ-related pathology for DS. The Ts65Dn mouse model of DS was selected due to its high face validity. The appropriate background strain was selected in accordance with the Jackson Laboratories instructions. All experiments were controlled both by a sham-treatment and wildtype controls. Vaccinated mice are indicated using the abbreviation /V (i.e. Ts65Dn/V), and sham-treated control mice using /C (i.e. Ts65Dn/C). Sample size was determined according to the convention in behavioral testing and was set to 12-randomly allocated mice per group. For all learning tasks, data collection concluded when no further improvement was observed for two consecutive days. All the behavioral paradigms were performed in a blinded manner at 3–4 different time points. Details of all behavioral testing are found in supplementary Table 1. The endpoint of the experiment was set to 15 months of age to ensure full neuropathological assessment prior to premature death due to trisomy (Davisson, 2005).

### 2.2. Animals

Ts(17^16^)65Dn, (Ts65Dn), a widely used mouse model for Down-syndrome, encompass a partial trisomy of Mmu16 and Mmu17, containing 92 genes orthologous to Hsa21 and over-expresses mouse APP and DYRK1A (Reeves et al., 1995; Rueda et al., 2012). Four weeks-old male Ts65Dn mice, and their appropriate background strain (B6EiC3Sn.BLiAF1/J) were purchased from the Jackson Laboratories (Bar Harbor, ME) (Ts65Dn JAX stock #005252, JAX stock B6EiC3Sn.-BLiAF1/J #003647). Animals were housed in a reversed 12:12hr cycle. Animal care and experimental procedures followed Bar Ilan University’s guidelines and were approved by the Bar Ilan University Animal Care and Use Committee.

### 2.3. Aβ-CoreS vaccine

Aβ-CoreS is a DNA vaccination based on the pVAX1 expression vector (Olkhanud et al., 2012), containing DNA coding the N-terminus-Aβ_1–11_ fused to a Hepatitis-B surface antigen (HBsAg), a central component of the hepatitis B virus (HBV). Aβ-CoreS also contains a T-helper epitope of a Hepatitis-B core antigen (HBcAg) which originates from the capsid antigen of HBV to facilitate antibody production (Pyrski et al., 2017). The Aβ-CoreS plasmid has a total length of ~3.8 Kb, with a vaccine construct length of 824 bp. As a control treatment, an expression vector (pUC19, New England Biolabs) containing HBsAg was used. This plasmid is ~4.2 Kb long, with a sham construct length of 680 bp.

### 2.4. DNA microcarriers and cartridges preparation

Gold particles (Bio-Rad Laboratories, Hercules, CA 94547, USA) were used as microcarriers for the high-helium-pressure delivery of DNA vaccination. Microcarrier loading quantity (MLQ), the amount of gold particles, was set to 0.5 mg per target according to the manufacturer’s recommendation for in vivo delivery. The amount of DNA loaded per mg of microcarriers is referred to as the DNA loading ratio (DLR), was set to 1 μg/mg DNA/gold particles, giving 0.5 mg of gold particles and 0.5 μg of DNA per cartridge. Cartridge preparation was conducted according to the manufacturer’s protocol (BIO-RAD Helios gene gun system instruction manual, #165–2431 and #165–243). Briefly, spermidine, CaCl_2_, polyvinylpyrrolidone solutions and gold particles were added to the purified DNA (at a concentration of 500 ng/μl) to bind it to the gold particles. The DNA-gold mixture was injected to plastic tubes and dried using a gentle stream of nitrogen gas. Gold-DNA coated tubes were cut into 1 cm-long cartridges. Cartridges were stored in a desiccator at 4 °C until use.

### 2.5. Vaccine administration

Immunization was administered 3 times starting at the age of 6 m at a dose of 1 μg of DNA per immunization episode. Mice abdomen skin was shaved, cleaned with ethanol (70%) and dried. DNA was delivered to the mice abdomen skin using the Helios gene gun (Bio-Rad Laboratories, Hercules, CA 94547, USA) attached to a compressed helium tank of grade 4.5 (> 99.995%), at 300 psi, according to manufacturer’s protocol.

### 2.6. Oligomeric murine Aβ peptide preparation

Recombinant rat/mouse Aβ_1–42_ peptide (#ab120959, Abcam, Cambridge, UK), was dissolved in 1,1,1,3,3,3-hexafluoro-propanol (HFIP), lyophilized and brought up in DMSO to 1 mg/ml according with manufacturer’s protocol. To induce oligomerization, monomeric Aβ solution was incubated in low-salt buffer (10 mM phosphate buffer, 10 mM NaCl, Ph 7.4) for 24 h at 4 °C (Ahmed et al., 2010).

### 2.7. Western blot for Ag-Ab interaction

Oligomeric murine-Aβ_1–42_ solution was diluted in SDS-PAGE sample buffer, boiled at 95 °C for 10 min and loaded to 15% (w/v) Tris-glycine polyacrylamide gels. Electrophoresed peptides were transferred to a PDVF (IPVH00010 Immobilon-P Membrane, PVDF, 0.45 μm, Mercury) membrane and blocked for unspecific binding using 5% skim milk diluted in 0.1% (v/v) PBS-Tween20 (P9416–50ML, Sigma, St. Louis, MO, USA). Next, serum from either Aβ-CoreS-vaccinated or naïve mice, diluted to 1:50 in blocking buffer were added for overnight incubation at 4 °C. Unbound antibodies were washed 3 times in PBS-T (0.1% Tween 20) for 5 min and membrane was incubated with HRP-conjugated goat anti mouse IgG secondary antibody (Cat# 115–035-003, Peroxidase AffiniPure Goat Anti-Mouse IgG, Jackson immunoreasearch, PA, USA) diluted at 1:10,000 in blocking buffer for 1 h at room temperature (RT). Ab-Ag bindings were detected by applying ECL (ECL kit, 20–500-120, Biological industries, Israel).

### 2.8. Antibody titer

Specific anti-Aβ_1–11_ antibody production was quantified by an indirect ELISA. 96-well high binding microplates (Microlon, 655061, Greiner bio-one, Monroe, NC, USA) were covered with 50 μl of recombinant mouse Aβ_1–42_ peptide solution (Amyloid-beta peptide (1–42) [mouse/rat], 120959, Abcam, Cambridge, UK) in carbonate/bicarbonate coating buffer (pH 9.6) to a concentration of 3 μg/ml. Plates were incubated overnight at 4 °C, washed 3 times with 0.1% PBS-Triton and blocked with 2% BSA (A7906, Sigma, St. Louis, MO, USA) for 1 h at RT. Plates were washed 3 times with PBS-T and incubated serum samples diluted at 1:100–1:12500 for 1 h at RT. Standard curve was carried out using known concentrations of primary goat-anti-mouse Aβ1–16 antibody (50–500 ng/ml, ab126873, Abcam, Cambridge, UK). Plates were washed 3 time in PBS-T and incubated with HRP-conjugated goat anti-mouse IgG secondary antibody diluted at 1:5000, (115–035-003, Peroxidase AffiniPure Goat Anti-Mouse IgG, Jackson immunoreasearch, PA, USA) or goat anti-rabbit secondary antibody of standard curve wells (111–035-003, Peroxidase AffiniPure Goat Anti-Rabbit IgG, Jackson Immunoresearch, PA, USA) for 1 h at RT. Plates were washed and 3,3′,5,5′-tetramethylbenzidine (TMB) substrate (00–4201-56, Affimetrix eBioscience, San Diego, CA, USA) was added. Colorimetric reaction was stopped by adding 50 μl of 2 M H_2_SO_4_ solution (339741, Sigma-Aldrich, St. Louis, MO, USA), and OD was measured at 450 nm using a spectrophotometer.

### 2.9. Immunoglobulin isotyping

To measure levels of specific anti Aβ IgG1, IgG2a, IgG2b, IgG3, IgM, and IgA, a similar indirect ELISA protocol was conducted with the addition of specific anti-mouse-immunoglobulin antibodies (ISO-2, Sigma-Aldrich, St. Louis, MO, USA) diluted at 1:1000, incubated for 1 h at RT. Next, donkey anti-goat secondary antibody (705–035-003, Peroxidase AffiniPure Donkey Anti-Goat IgG, Jackson Immunoresearch, PA, USA) diluted at 1:5000 was applied for 1 h at RT. Colorimetric reaction was stopped by adding 50 μl of 2 M H_2_SO_4_ solution (339741, Sigma-Aldrich, St. Louis, MO, USA), and OD was measured at 450 nm using a spectrophotometer.

### 2.10. Elevated zero maze

Anxiety was monitored using the elevated zero maze (EZM), a ring-shaped 65 cm-high table, divided into closed and opened sections. The ring is 7 cm wide and has an outer diameter of 60 cm. The closed sections are confined by 20 cm-high walls and a semi-transparent ceiling, whereas the opened sections have a 0.5 cm high curbs at the edges, to prevent animals from falling. Illumination was kept at 1300 lx and trial duration was 5 min. Animal presence in the open/closed sections of the elevated zero maze were monitored using video tracking.

### 2.11. Open field

Exploratory behavior was quantified using the Open field test a 40 × 40 cm square arena. The outer 8 cm were defined as the area periphery, and the 24 × 24 cm inner square as the center. Illumination was kept at 1300 lx. Mice were allowed to freely explore the arena for 5 min.

### 2.12. Radial arm water maze

Spatial learning capacity was tested using the radial arm water maze (RAWM), constructed of a 150 cm of diameter pool, with eight 10 cm-wide, 60 cm-long radial arms, and a 12 × 12 cm platform located at the end of one arm. Water was kept opaque using white non-toxic paint, at a constant temperature of 27 ± 0.5 °C and illumination of ~20 lx. For habituation, mice were given 90 s to find a visible platform, 3 trials a day, for four consecutive days. Animals that did not manage to locate the target were put on the platform by the experimenter. All animals had 60 s of resting on the platform.

In the acquisition phase, mice were required to search for a hidden platform located 1.5 cm underneath water line. Four different visible extra-maze cues were presented the walls, at equal distances from its center. Mice were placed in the central zone of the maze and were allowed 90 s to find the platform. This trial repeated 3 times daily, until no significant improvement in performance was identified. Twenty-four hours following acquisition, a probe test was conducted, in which the platform was removed from the pool. Mice were given 60 s and one trial to explore the pool.

### 2.13. Barnes maze

Spatial learning capacity was assessed using the Barnes maze (Barnes, 1979; Barnes et al., 1980; Bimonte-nelson; Bonaccorsi et al., 2013; Illouz et al., 2016a), a circular table, 105 cm high and a diameter of 92 cm. Eighteen holes are located at the perimeter of the table at equal distances, each with a diameter of 5 cm. One hole (the target hole) leads to an escape chamber in which the animal can hide. Illumination was measured at the center of the table and maintained at 1300 lx to encourage the animals’ motivation to search for the target hole. During the habituation phase, which lasted one day, the animal was placed in a cylinder at the center of the maze. Five seconds later, the cylinder was removed, and the mouse was allowed to explore the environment for 120 s. Mice that found the target hole were able to enter the escape chamber; mice that did not find it within this period of time were placed back in the cylinder, now located above the target hole. In this phase mice were given one trial only. Four extra-maze visual cues were presented on the walls surrounding the Barnes table. In the spatial acquisition phase, mice were given 120 s per trial to find the target hole, for three trials per day, with an inter-trial interval of 10 min. This procedure was repeated daily until no significant improvement in performance was identified. Following spatial acquisition, a probe test was conducted with closed holes and no escape chamber. Animals were given a single 60 s to explore the environment. Following the probe test, the target hole and escape chamber were rotated 180° from the original target location. Similar to the spatial acquisition phase, mice were given 3 non-sequential trials, 120 s each, to find the new escape chamber.

### 2.14. Spatial strategy analysis

Spatial search strategies in the Barnes maze were analyzed using the BUNS algorithm (Illouz et al., 2016a; Illouz et al., 2016b). In brief, a support vectors machine (SVM) (Boser et al., 1992; Vapnik, 1998) classifier was applied to classify performance of mice in the Barnes task into spatial strategies to quantify their cognitive capacity at a higher resolution.

### 2.15. T-maze

We utilized a variant of the T-maze spontaneous alternation test modified from (Deacon and Rawlins, 2006). Briefly, T-maze arms were 30 cm long and 15 cm wide, walls were 15 cm high, covered by different black and white patterns. Mice were given 3 trials with an intertrial interval of 2hrs, each trial consisted of 2 stages: During acquisition, mice were released from the starting chamber and were given the opportunity to enter one of the target arms. A trial was ended when the animals spent more than 2 s with all 4 limbs inside one of the target arms. Next, mice were allowed to stay in the chosen arm for 30 s, followed by a repetitive trial in which alternation rate was measured.

### 2.16. Novel object recognition test

Short-term memory was assessed using the novel object recognition (NOR) test as previously described (Leger et al., 2013). In short, mice were placed in a 40 × 40 cm arena with two different objects. In the first trial, mice were allowed to explore their environment for 10 min. In the second trial, one of the objects was replaced by a visually different object. Time spent near the two objects was measured as animals with intact short-term memory are expected prefer the novel object.

### 2.17. Serum collection

Blood was extracted from the facial vein using a glass cannula and incubated for 30 min at RT to clot. Samples were then centrifuged at 1000×g for 8 min at 4 °C. Clear serum was stored at −20 °C for further analysis.

### 2.18. Brain sample collection

Mice were anesthetized using Ketamine-Xylazine (100 mg/kg, Vetoquinol, France, 10 mg/kg, Eurovet, The Netherlands, respectively) and perfused with PBS. Hemibrains were then separated for histological and biochemical analysis. For Histology, hemibrains were transferred to 4% paraformaldehyde (PFA) and stored at 4 °C for 48 h. Following fixation, tissues were transferred to a gradient of 20% and 30% sucrose aqueous solutions for 24 h each. Hemibrains were then dissected into 40 μm-thick slices using a microtome and stored in a cryoprotectant solution (containing 30% glycerol and 35% ethylene glycol) at −20 °C until use. For biochemical analysis, cerebral cortex and hippocampi were separated, snap-froze on dry ice and stored at −80 °C until use. For Aβ quantification using ELISA, soluble and insoluble protein fractions were purified using our previously published protocol (Illouz et al., 2017). Briefly, tissues were mechanically homogenized in 0.1% SDS-RIPA buffer (150 mM NaCl, 5 mM EDTA, 50 mM Tris-base, 1% NP-40, 0.5% Na-deoxycholate, 0.1% SDS in aqueous solution), 15 μl per 1 mg tissue, incubated on ice for 30 min followed by centrifugation for 90 min at 17,000g at 4 °C. Supernatant, containing RIPA-soluble fraction of mouse Aβ_1–40_ and Aβ_1–42_ was removed and stored at −20 °C. The centrifuged pellet, containing the insoluble fraction was incubated for 30 min in Trifluoroaceticacid (TFA, > 99%, 200–929-3, Sigma-Aldrich, St. Louis, MO) at RT. Next, samples were dried under a gentle stream of nitrogen gas to 10% of the original volume, re-suspended in PBS, neutralized with 1 N NaOH and centrifuged again at 17,000×g for 90 min at 4 °C. The supernatant, containing RIPA-insoluble fraction was removed and stored at −20 °C. Total protein concentration was determined using the BCA method (Cat# 23225, ThermoFisher Scientific, Waltham, MA, USA).

### 2.19. Thioflavin-S staining

β-Sheet conformations were detected using a Thioflavin-S staining protocol, adapted from Rajamohamedsait and Sigurdsson (2012). In brief, 40 μm-thick brain sections were rinsed in 0.1% PBS-Triton and incubated with 1% Thioflavin-S aqueous solution for 15 min. Sections were dehydrated through incubation in serial ethanol solutions: 70%, 80%, 95% and 100% for 2 min each.

### 2.20. Measuring Aβ40/42 levels using sELISA

Soluble and insoluble levels Aβ_1–40_ and Aβ_1–42_ in the serum and brain-tissue were conducted using our previously published sandwich-ELISA protocol (Illouz et al., 2017). In brief, 96-well polystyrene microplates (Microlon, 655061, Greinerbio-one, Monroe, NC) were covered with 50 μl of anti-mouse-N-terminus Aβ_1–16_ (ab126873, Abcam) at a concentration of 5 μg/ml in carbonate-bicarbonate buffer (pH = 9.6) and incubated overnight at 4 °C. Plates were washed 5 times in PBS-T solution (0.1% Triton-x in PBS) and blocked with 2% BSA solution in PBS. 50 μl of serum or tissue homogenate were applied to each well, and incubated for 90 min at RT. Plates were then washed 5 times in PBS-T, and the following detection antibodies were added: Anti-Aβ_1–40_ antibody (ab20068, Abcam) diluted at 1:500 or anti Aβ_1–42_ antibody (05–831, Millipore, Billerica) at 1:2500, and incubated for 90 min at RT. Next, plates were washed 5 times in PBS-T and secondary goat-anti-mouse IgG HRP-conjugated antibody was added (115–035-003, Peroxidase AffiniPure Goat Anti-Mouse, Jackson immunoreasearch, PA, USA) at a dilution of 1:5000. Plates were washed 5 times with PBS-T and 50 μl of 3,3′,5,5′-tetramethylbenzidine (TMB) substrate (00–4201-56, Affimetrix eBioscience, San Diego, CA, USA) was added. The color reaction was allowed to develop for 3 min and was stopped by adding 50 μl of 2 M H2SO4. Optical density (OD) was measured at 450 nm using a spectrophotometer.

### 2.21. Measuring p-Ser396-tau levels using sELISA

A similar sELISA protocol was applied for the quantification of phosphor-Serine-396 tau protein in the cortex and hippocampus. We used chicken-anti-tau (ab75714, Abcam, Cambridge, UK) as coating antibody and rabbit-anti-phosphor-ser396-tau (ab109390, Abcam, Cambridge, UK) as detection antibody.

### 2.22. Immunohistochemistry

40 μm-thick hemibrains were rinsed 5 times in 0.1% PBS-Triton for 5 min. Nonspecific bindings were blocked using 20% normal horse serum in PBS-T for 1 h at RT. For Aβ staining, antigen retrieval was conducted using 75% formic acid for 2 min, at RT. Primary antibody was then applied and incubated overnight at 4 °C. The following primary antibodies were used: mouse-anti-Aβ_1–40_ (ab20068 Abcam, Cambridge, UK) diluted at 1:200, mouse-anti-Aβ_1–42_ (05–831 Millipore, Billerica, MA) diluted at 1:2500, mouse-anti-NeuN (MAB377, Billerica, MA) diluted at 1:10,000, rabbit-anti-Iba1 (019–19741, WAKO, Japan) diluted at 1:1000, rat-anti-CD68 (ab53444, Abcam, Cambridge, UK) diluted at 1:2500, rat-anti-Clec7a (mabg-mdect, Invivogen, San Diego, CA) diluted at 1:50, rat-anti-4D4 and rabbit-anti-P2RY12, generously provided by O. Butovsky (Ann Romney Center for Neurologic Diseases, Department of Neurology, Brigham and Women’s Hospital, Harvard Medical School, Boston, MA 02115), diluted at 1:4000, 1:200, respectively. Astrocytes were visualized using a rabbit-anti-GFAP antibody (M0761, Agilent, Santa Clara, CA) diluted at 1:7,500, rabbit-anti-S100β (ab52642, Abcam, Cambridge, UK) diluted at 1:7500 and Rat-anti-C3 (ab11862, Abcam, Cambridge, UK) diluted at 1:50. For all astrocytic staining, antigen retrieval was performed by incubating brain slices in citrate buffer (pH = 6) for 20 min. Next, sections were rinsed 5 times in PBS-T for 5 min and fluorescence-tagged secondary antibodies were applied for 1 h at RT: Goat-anti-Mouse IgG (Alexa-488/568, 1:1000, Invitrogen, OR, U.S.A.), Goat-anti-Rabbit IgG (Alexa-488/568, 1:1000, Invitrogen), Goat-anti-Rat IgG (Alexa-488/568, 1:1000, Invitrogen). Slices were then stained with Hoechst 33,342 (H3570, Invitrogen) diluted at 1:1000.

### 2.23. Stereological analysis of cells in the CNS

#### 2.23.1. Microglial and astroglial cells

The hippocampus was outlined according to the Paxinos atlas of the mouse brain. Quantification of stained cells was evaluated by stereological counts using the optical dissector method as described previously (West et al., 1991). Optical fractionator sampling was carried out using a Leica DM6000 microscope (Leica Microsystems, Germany) coupled to a controller module and a high-sensitivity 3CCD video camera system (MBF Biosciences, VT), and an Intel Xeon workstation (Intel, CA). Sampling was implemented using the Stereo Investigator software (MBF Biosciences). Analyzed brain sections spanned from −0.94 mm to −4.04 mm from Bregma. Every 9th to 10th section (360–400 μm apart) was used for quantification from each animal. Counting frame size was set to 100 × 100 μm. The total number of positive cell population was estimated based on the section volume and extrapolated for the total volume of the hippocampus. An experimenter blind to all treatment groups performed the stereological counts. Microglial number of branches per cell and branches complexity were assessed using the WIS-NeuroMath algorithm (Rishal et al., 2013). Image fluorescence intensity was calculated per pixel, filtered for noise reduction and normalized to the number of cells using MATLAB (MathWorks, Natick Massachusetts).

#### 2.23.2. Neuronal cells

The retrosplential cortex was outlined according to the Paxinos atlas of the mouse brain. Analyzed brain sections spanned from −1.35 mm to −2.05 mm from Bregma. Quantification of stained cells was evaluated by stereological counts as described above, with counting frame size was set to 50 × 50 μm. Calculation of total number of cells was conducted as describe above.

### 2.24. Statistical analysis

The data presented as mean ± SEM were tested for significance in the unpaired t-test with equal variances, one-way ANOVA, repeated-measures (RM) two-way ANOVA, two-sample Kolmogorov-Smirnov test, Pearson’s correlation coefficient or the χ^2^ test for independence. post-hoc tests were conducted using the Tukey or Bonferroni corrections. All error bars represent SEM were calculated as $\frac{std(\text{x})}{\sqrt{\text{n}}}$ for numeric variables, and as $\sqrt{\frac{\text{p}(1-\text{p})}{\text{n}}}$ for binomial variables. Outliers were identified using the Robust regression and outlier removal (ROUT) method with coefficient Q = 1% (Motulsky and Brown, 2006). Significant results were marked according to conventional critical P values: *P < 0.05, **P < 0.01, ***P < 0.001, ****P < 0.0001.

### 2.25. Data availability

All the data support the findings of this study are available from the corresponding author upon request.

## 3. Results

### 3.1. Ts65Dn mice exhibit reduced cognitive capacity

To assess the efficacy of the AβCore-S vaccination in the Ts65Dn DS mouse model, we first conducted a baseline behavioral assessment on Ts65Dn and WT mice at 3 m of age prior to immunization (*n* = 24 per group, Fig. S1A). Ts65Dn mice exhibited a higher fraction of time spent in the open arms of an elevated zero maze compared with WT mice (0.37 ± 0.02 and 0.26 ± 0.01 respectively, P < 0.001, Fig. S1B), suggesting higher anxiety threshold in these mice. While covered distance (Fig. S1C, P > 0.05), movement speed (Fig. S1D, P > 0.05) and number of zone crosses between the open and closed arms (Fig. S1E, P > 0.05) did not differ in the elevated zero maze, covered distance (P < 0.01, Fig. S1F) and mean speed (P < 0.01, Fig. S1G) were higher among Ts65Dn mice compared with WT mice in the open field arena. Despite this, no strain differences were observed in time spent in the corners, periphery or center zones of the open field (P = 0.59, Fig. S1H), suggesting that the exploratory behavior is intact at the age of 3 m. These data are consistent with previous reports of a motor-hyperactivity in the Ts65Dn mice (Faizi et al., 2011). To obtain a baseline for hippocampal-dependent spatial capacity, mice were initially tested using the radial arm water maze. However, our observations indicate that young Ts65Dn mice are severely impaired in this task. Latency to reach the platform and total distance travelled were dramatically higher in Ts65Dn mice throughout the acquisition phase (latency: 41.36 ± 3.56 s and 9.34 ± 0.86 s, respectively, P < 0.0001, Fig. S2A; distance: 3.49 ± 0.34 m and 1.29 ± 0.13 m, respectively, P < 0.01, Fig. S2B. Data relates to the last acquisition day). Accordingly, Ts65Dn mice exhibited lower path efficiency to the platform (0.35 ± 0.03 and 0.71 ± 0.3 respectively, P < 0.0001, at the last day of acquisition, Fig. S2C). Swimming speed of Ts65Dn mice was significantly lower compared with WT mice (0.08 ± 0.004 and 0.14 ± 0.003 m/s, respectively, P < 0.0001, Fig. S2D). Additionally, reference memory (RM) error rate at the last day of the radial arm water maze acquisition task was higher in Ts65Dn mice compared with WT mice (2.1 ± 0.21 and 0.65 ± 0.11 errors, respectively, P < 0.0001, Fig. S2E, G). However, while working memory (WM) error rate was initially higher in Ts65Dn mice, there was no significant difference between the strains by the last day of acquisition (P = 0.18, Fig. S2F, G).

Since we established that Ts65Dn mice exhibit an inherent deficit in the radial arm water maze swimming task, we further assessed the spatial learning capacity of Ts65Dn mice in the Barnes maze, a non-water-based task that assesses spatial learning (Fig. S3A). Latency to reach the target hole did not differ between Ts65Dn and WT mice (P = 0.98, Fig. S3B), however the distance travelled was significantly higher in the Ts65Dn group on days 2–4 (P < 0.01, P < 0.0001 and P < 0.05 respectively, Fig. S3C). In addition, the mean traveling speed of Ts65Dn mice was higher on acquisition days 2–7 (P < 0.05 on day 2, P < 0.0001 on days 3–7, Fig. S3D), and their path efficiency was lower (0.46 ± 0.03 and 0.64 ± 0.03 on the last day, P < 0.001, Fig. S3E). Elevated speed and lowered path efficiency along with equal latencies reflects a lower spatial memory acquisition in the Ts65Dn strain, as these mice compensate their lack of orientation with traveling at a higher speed (Fig. S3B-E). RM errors were higher in Ts65Dn mice (P < 0.0001, Fig. S3F), while WM error rates did not differ between strains (P = 0.16, Fig. S3G), suggesting that Ts65Dn mice may also compensate their reduced spatial capacity by numerous entries to random holes. To confirm this, we performed a spatial strategy analysis with the Barnes maze UNbiased Strategy classification (BUNS) algorithm (Illouz et al., 2016a). The BUNS analysis revealed that while WT mice mostly used the *direct* and *corrected* search strategies (30.5 and 25% respectively, Fig. S3H, left panel) on the last day of acquisition, Ts65Dn mice used these strategies at a lower rate (16.67 and 24.24% respectively, Fig. S3H, right panel) with *serial search* as the prominent strategy (28.78%, Fig. S3H, right panel). Importantly, the cognitive scores of Ts65Dn mice, reflecting their ‘spatial IQ’, were significantly lower throughout the acquisition task compared with WT mice (0.61 ± 0.03 and 0.78 ± 0.027 respectively, P < 0.01, Fig. S3I). Qualitative presentation of mice’ spatial location using heat maps and trajectory plots reveals a prolonged period spent in the center of the maze at the beginning of trials followed by a direct approach to the target hole by WT but not Ts65Dn mice (Fig. S3J). In the probe test, both strains exhibited a Gaussian-like distribution of hole-entries centered around the target hole (Fig. S3K, left and middle panels). As a result, the difference in entropy (Δ entropy) between these distributions and a theoretical uniform distribution (yielding the maximal entropy) were similar (Fig. S3K, right panel).

We next assessed memory retention in a reversal task of the Barnes maze, which presents a higher difficulty level under identical external spatial cues. Latency to reach the new target was surprisingly lower in Ts65Dn mice in days 2–4 compared with WT mice (20.52 ± 1.62 and 30.43 ± 1.86 s, respectively, on the last day, P < 0.05, Fig. S4A), suggesting effective long-term spatial plasticity in Ts65Dn mice. With the exception of the first day (4.89 ± 0.52 m for DS, 2.44 ± 0.14 m for WT, P < 0.0001, Fig. S4B), distance did not differ between strains (P = 0.52, Fig. S4B), however Ts65Dn mice travelled at a higher speed than WT mice throughout the experiment (0.1 ± 0.007 m/s and 0.05 ± 0.003 m/s, respectively, on the last day, P < 0.0001, Fig. S4C). Path efficiency, RM and WM error rates did not differ in the reversal task (P > 0.05, Fig. S4D-F respectively). BUNS analysis revealed that although WT mice exhibited *direct* and *corrected search* strategies at higher rate than Ts65Dn mice (34 and 18% (combined), respectively, Fig. S4G) the most prevalent strategies among both strains was *serial search* (54 and 51%, respectively, Fig. S4G), reflecting a higher cognitive demand in the reversal than in the acquisition task. Although higher in the WT group, cognitive scores did not significantly differ in this task (P = 0.058, Fig. S4G, right panel). Heat maps and trajectory plot support the strategies indicated by the BUNS analysis (Fig. S4H).

The baseline cognitive assessments indicate that Ts65Dn mice exhibit (a) higher anxiety threshold, (b) motor hyper activity and elevated travelling speed (c) motor deficit in swimming tasks and (d) cognitive impairment in spatial learning tasks, compared with the WT group (Figs. S1–S4).

### 3.2. Ts65Dn mice vaccinated with AβCore-S generate Aβ-specific IgM and IgG responses

**Fig. 1.**
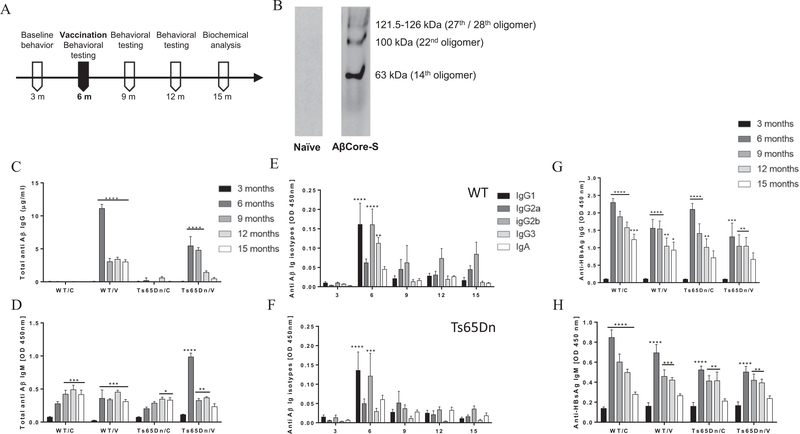
Vaccination with AβCore-S DNA vaccine at 6 months induces anti-Aβ IgM and IgG production in Ts65Dn and WT mice with high affinity to oligomers of murine Aβ. (A) Ts65Dn and WT mice were vaccinated at the age of 6 m with either AβCore-S or sham treatment for three times in a 14d interval. Total IgG, IgM and IgA titers as well as IgG subclasses were assessed every 3 months using indirect ELISA. (B) Antibodies found in the sera of AβCore-S-vaccinated mice effectively bind various Aβ42 oligomers (63,100,121.5–126 kDa), as assessed in western blot, compared to naïve serum (C) Total anti-Aβ IgG antibodies were significantly increased after immunization in both Ts65Dn (P < 0.0001) and WT (P < 0.0001) compared to baseline. Antibody levels remained high in the age of 12 m in Ts65Dn mice (P < 0.05) and 15 m (P < 0.001) in WT mice (D) Vaccinated Ts65Dn mice produce a higher IgM titer compare with their base line as well as with vaccinated WT mice. IgM levels remain high in this group until the age of 15 m. However, an age-dependent elevation in IgM levels was observed in both control groups. (E) IgG subclasses and IgA levels over time in vaccinated WT mice. IgG1 (P < 0.0001), IgG2b (P < 0.0001) and IgG3 (P < 0.001) were higher immediately after immunization and decreased to baseline by 9 m (F) IgG subclasses and IgA levels over time in vaccinated Ts65Dn mice. IgG1 (P < 0.0001) and IgG2b (P < 0.001) were higher immediately after immunization and decreased to baseline by 9 m (G) Anti-HBsAg IgG antibody levels increased in all groups immediately after immunization (P < 0.0001) and remained at high concentration until the age of 12 m for both Ts65Dn groups (P < 0.01) and 15 m for the WT/C, WT/V groups (P < 0.001, P < 0.05, respectively) (H) Anti-HBsAg IgM antibody levels increased in all groups immediately after immunization (P < 0.0001) and remained at high concentration until the age of 12 m for both transgenic groups (P < 0.01) and WT/C, WT/V groups (P < 0.0001, P < 0.001, respectively). Repeated-measures two-way ANOVA. *P < 0.05, **P < 0.01, ***P < 0.001, ****P < 0.0001.

At 6 m of age, Ts65Dn and WT mice were immunized using a Helios gene-gun with the AβCore-S vaccine, containing Aβ_1–11_ fused to the HBsAg epitope. A vaccine construct containing the HBsAg component alone served as sham-control treatment. Mice were vaccinated three times with 14d intervals (Fig. 1A). To characterize vaccine-induced antibodies, blood was collected at 12 days following the last boost and was used for the detection of oligomeric murine Aβ_1–42_ in western blotting. Antibodies found in the sera of vaccinated WT mice, effectively bound Aβ_1–42_ oligomers (63 kDa, 100 kDa, 121.5–126 kDa, Fig. 1B). In contrast to most AD transgenic mouse strains that encompass a mutated human APP, PS1 or PS2, TsDn65 mice expresses an extra copy of the endogenous murine APP gene. Cross reactivity of the human and murine variants of the AβCore-S vaccine was tested with recombinant murine and human Aβ_1–42_ (m/hAβ) using ELISA. No cross reactivity between hAβ_1–42_ peptide to antibodies against mAβ_1–11_, or between mAβ_1–42_ peptide and antibodies against hAβ_1–11_ were detected (Fig. S5A, B, respectively), suggesting high specificity of the AβCore-S vaccine.

Following immunization, at the age of 6 m, specific anti-Aβ IgG serum levels were measured using ELISA. Antibody titer of Ts65Dn mice peaked at 5.45 ± 1.4 μg/ml, and remained high until 9 m of age (P < 0.0001, Fig. 1C). Vaccinated WT mice produced higher IgG levels compared with Ts65Dn (11.12 ± 0.62 μg/ml and 5.45 ± 1.4 μg/ml respectively; P < 0.0001, Fig. 1C), which remained high until 15 m. Importantly, specific anti-Aβ IgM levels were higher in vaccinated Ts65Dn mice immediately after immunization, compared with vaccinated WT mice (0.98 ± 0.05 and 0.35 ± 0.12, respectively, P < 0.0001, Fig. 1D). IgM levels remained high until 12 m in Ts65Dn mice (P < 0.01, Fig. 1D) and 15 m in WT mice (P < 0.001, Fig. 1D). Interestingly, an age-dependent elevation of anti-Aβ IgM was found in the serum of sham-vaccinated WT mice at 9 m of age (P < 0.001, Fig. 1D) and in 12 m-old sham-vaccinated Ts65Dn mice (P < 0.05, Fig. 1D). To further characterize the humoral immune response elicited by the AβCore-S vaccine, we measured levels of Aβ-specific IgG1, IgG2a, IgG2b, IgG3 and IgA isotype levels. IgG1 and IgG2b were the most prevalent isotypes among both WT (P < 0.0001, Fig. 1E) and Ts65Dn mice (P < 0.0001 and P < 0.001 respectively, Fig. 1F) immediately after immunization. In WT but not Ts65Dn mice, high IgG3 antibodies were also present in the serum at 6 m (P < 0.01, Fig. 1E). IgG2a were present in both strains with no significant differences (P = 0.24, Fig. 1E, F), and no detectable IgA production was observed (P = 0.97, Fig. 1E, F).

Since an age-dependent decrease in Aβ-Specific antibody level was observed, anti-HBsAg IgG and IgM production was also measured (Fig. 1G, H, respectively). Post immunization, anti HBsAg-IgG levels increased to a range of 1.3–2.3 OD, (P < 0.001, Fig. 1G) and remained high until 15 m of age in WT mice (range of 0.93–1.23 OD, P < 0.05, Fig. 1G) and 12 m in Ts65Dn mice (range of 1.01–1.04 OD, P < 0.01, Fig. 1G). A similar effect was found for HBsAg-specific IgM, which peaked at 6 m of age (range of 0.5–0.84 OD, P < 0.0001, Fig. 1H) and remained elevated until 12 m across all experimental groups (range of 0.39–0.49 OD, P < 0.01, Fig. 1H). These results indicate integrity of the humoral immune response of Ts65Dn mice.

### 3.3. Vaccination with AβCore-S ameliorates long-term spatial memory impairments in Ts65Dn mice

Following immunization, mice were tested repeatedly in a variety of behavioral and cognitive tasks (Sup. Table 1, Fig. S6A). To test exploratory behavior post-vaccination, mice were tested in the open field arena. Sham-vaccinated Ts65Dn mice travelled a longer distance compared with WT mice (22.52 ± 2.88 m and 11.96 ± 0.71 m, respectively, P < 0.01, Fig. S6B). No such effect was found between vaccinated Ts65Dn mice and their WT controls (P = 0.19, Fig. S6B). This is possibly due to reduced motor hyper-activity in vaccinated mice, as sham-vaccinated Ts65Dn mice travelled at a higher speed compared to controls (0.07 ± 0.009 and 0.04 ± 0.002 m/s, respectively, P < 0.01, Fig. S6C). Time spent in the corner, periphery and center of the open field arena did not differ between groups (P = 0.96, Fig. S6D), suggesting that exploratory response is intact in 6 m-old Ts65Dn mice. Anxiety assessment using the elevated zero maze revealed a strain but not a treatment effect, as both vaccinated and sham-vaccinated Ts65Dn mice exhibited a greater fraction of time spent in the open zones compared with WT controls (0.34 ± 0.04, 0.34 ± 0.02 for Ts65Dn mice, respectively, 0.24 ± 0.02, 0.18 ± 0.01 for WT, respectively, P < 0.0001, Fig. S6E), suggesting higher anxiety threshold in Ts65Dn mice. No difference in open-close zone-crossing was observed between groups (P = 0.48, Fig. S6F). Strain effects were also found for distance and speed as Ts65Dn travelled a longer distance at a higher speed compared with WT mice (P < 0.05, Fig. S6G, H).

**Fig. 2.**
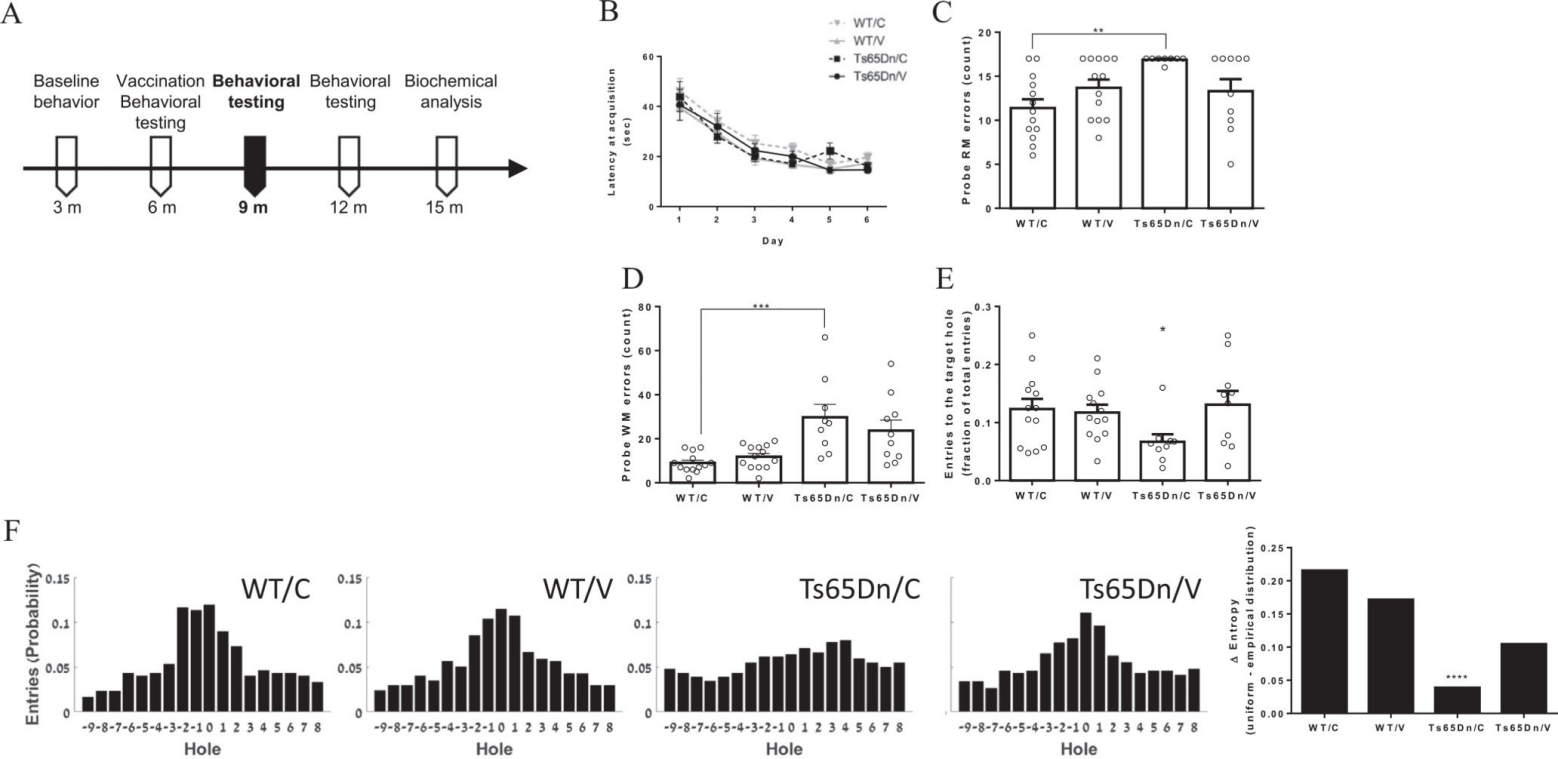
Vaccinated Ts65Dn mice obtain better results at the probe test of Barnes maze at 9 m of age. (A) Spatial learning capacity was assessed after immunization at 9 m of age using the Barnes maze. In the spatial acquisition phase (B) no difference was found for latency to reach the target between vaccinated, sham-vaccinated Ts65Dn mice and WT groups (P = 0.2). However, (C) sham treated Ts65Dn mice exhibit higher RM errors compared with sham-vaccinated WT mice (P < 0.01). (D) Ts65Dn mice from both groups showed a higher number of WM errors compared with WT mice (P < 0.05, P < 0.01, P < 0.001). (E) The fraction of target entries out of total hole-entries was lower for the sham-vaccinated Ts65Dn mice compared with all other groups (P < 0.05). (F) Distribution of hole-entries in the probe test of Barnes maze was closer to uniform in the sham-vaccinated Ts65Dn mice compared to all other groups, resulted in a lower Δ entropy (uniform – empirical distributions, P < 0.05). Repeated-measures two-way ANOVA, One-way ANOVA, Two-sample Kolmogorov-Smirnov test. *P < 0.05, **P < 0.01, ***P < 0.001, ****P < 0.0001.

We next tested the spatial learning capacity of the mice using the Barnes maze at 9 m (Fig. 2A). Similarly to their performance at the age of 3 months, latency to reach the target and travel distance did not differ between groups (P = 0.2, Fig. 2B and P = 0.12, Fig. S7A, respectively). Both Ts65Dn groups exhibited elevated speed and lower path efficiency compared with WT mice (P < 0.01, Fig. S7B and P < 0.01, Fig. S7C), suggesting that spatial accuracy is impaired in these mice due to hyperactivity.

RM but not WM errors were elevated in Ts65Dn mice compared to WT mice throughout the acquisition phase (P < 0.01 and P = 0.38, Fig. S7D, E, respectively). Spatial strategy analysis revealed that while on the last day of acquisition Ts65Dn mice utilized *direct* and *corrected* strategies at a rate of 17%, WT mice used these strategies in 38.5% of the trials (Fig. S7F). This was reflected in lower cognitive score in Ts65Dn mice by the fifth and last days (0.3–0.35 and 0.52–0.53, respectively, P < 0.05, Fig. S7F, heat maps and trajectory plots in Fig. S7G).

In the probe test, sham-vaccinated Ts65Dn mice exhibited higher number of RM errors compared with sham-vaccinated WT mice (16.8 ± 0.12 and 11.38 ± 1, P < 0.01, Fig. 2C), while vaccinated Ts65Dn mice showed no such elevation (P = 0.99, Fig. 2C). A similar effect was observed in working memory errors between sham-vaccinated Ts65Dn and sham-vaccinated WT mice (29.7 ± 5.82 and 9 ± 1.2, respectively, P < 0.001, Fig. 2D). Interestingly, these mice also exhibited a lower fraction of entries to the target hole compared with all other groups (P < 0.05, Fig. 2E). Distribution of entries to holes in the Barnes table was near-Gaussian for vaccinated Ts65Dn mice and both WT groups, and near-uniform for sham-vaccinated Ts65Dn (P < 0.0001, Fig. 2F). This is reflected in a lower difference of entropies between the empirical distribution and a theoretical uniform distribution, compared to all other groups (Fig. 2F). This finding suggests that vaccinating Ts65Dn mice with AβCore-S can ameliorate the spatial memory decline found in the Ts65Dn strain.

### 3.4. Vaccination with AβCore-S prevents short-term memory decline in Ts65Dn mice

**Fig. 3.**
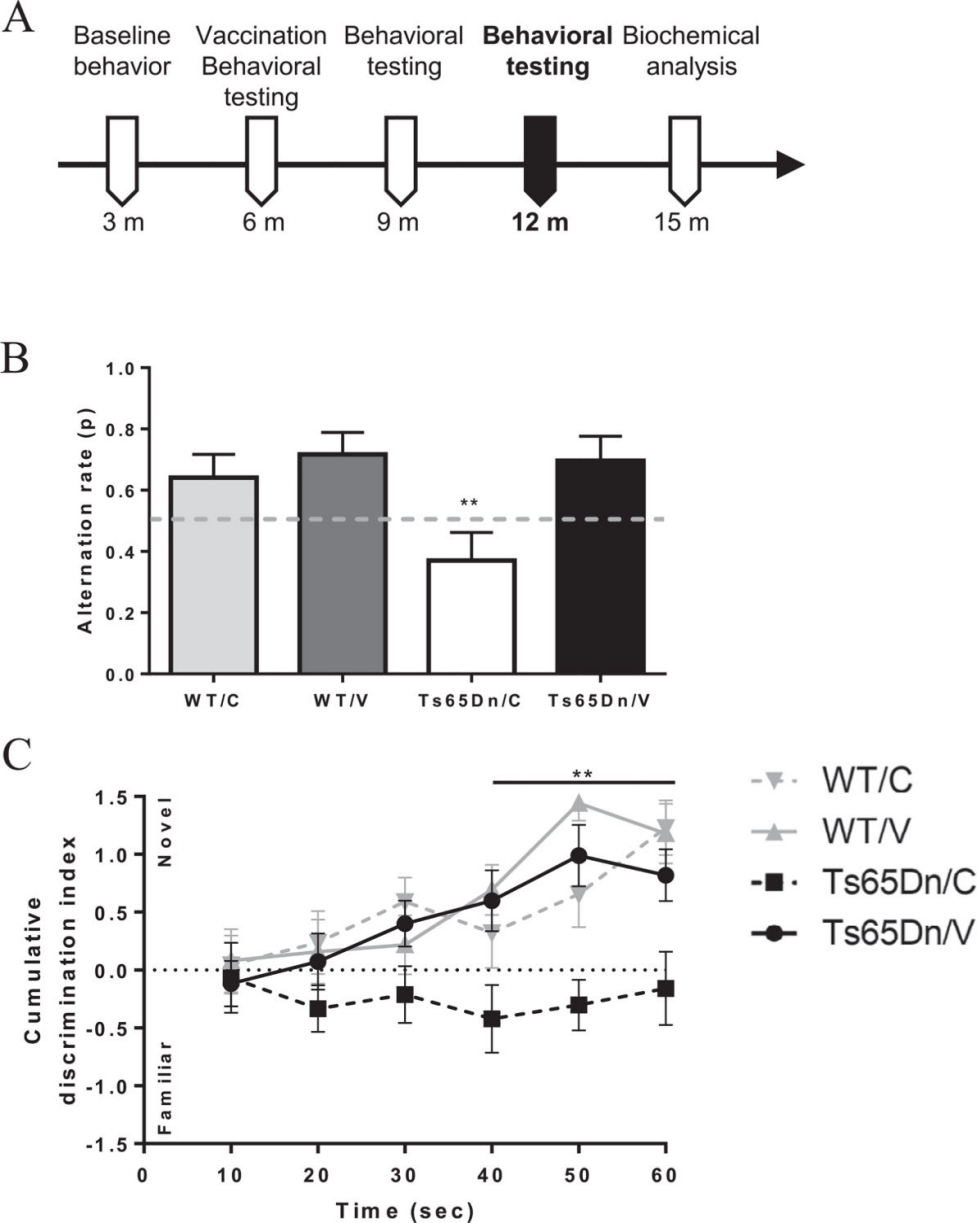
Immunization of Ts65Dn mice with AβCore-S rescues short-term memory. (A) Short-term memory was assessed at 9 m of age using the spontaneous-alternation T-maze and the Novel object recognition test. (B) Vaccinated Ts65Dn mice exhibit a higher alternation rate at the T-maze compared with sham-vaccinated Ts65Dn mice and at a similar level to both WT groups (P < 0.01). (C) Vaccinated Ts65Dn mice showed clear preferences to the novel object as indicated by cumulative discrimination index, compared to sham-vaccinated Ts65Dn mice and in a similar manner to both WT groups (P < 0.01). Chi-squared test for independence, Repeated-measures Two-way ANOVA, **P < 0.01.

Next, mice were tested for short-term memory using the spontaneous-alternation T-maze and the novel object recognition test (Fig. 3A). At 12 m, sham-treated Ts65Dn mice were outperformed in the T-maze task by vaccinated Ts65Dn mice WT controls (0.38 ± 0.08 and 0.64–0.71 ± 0.076, respectively, P < 0.01, Fig. 3B). The alternation rate of vaccinated Ts65Dn mice was similar to that of WT mice (0.69 ± 0.08 and 0.71 ± 0.07, respectively, P = 0.99, Fig. 3B). Consistent with these results, a lower cumulative discrimination index was found in sham-vaccinated Ts65Dn mice throughout the course of the novel object recognition test, compared with all other groups (−0.15 ± 0.31, 0.8–1.17 ± 0.23, at 60 s, respectively, P < 0.01, Fig. 3C). Vaccinated Ts65Dn mice however, performed normally in this task. These data indicate that short-term memory was rescued in vaccinated Ts65Dn mice.

### 3.5. AβCore-S reduces Aβ1–40 and Aβ1–42 serum levels and promotes clearance of Aβ1–40 and Aβ1–42 from the brain in Ts65Dn mice

**Fig. 4.**
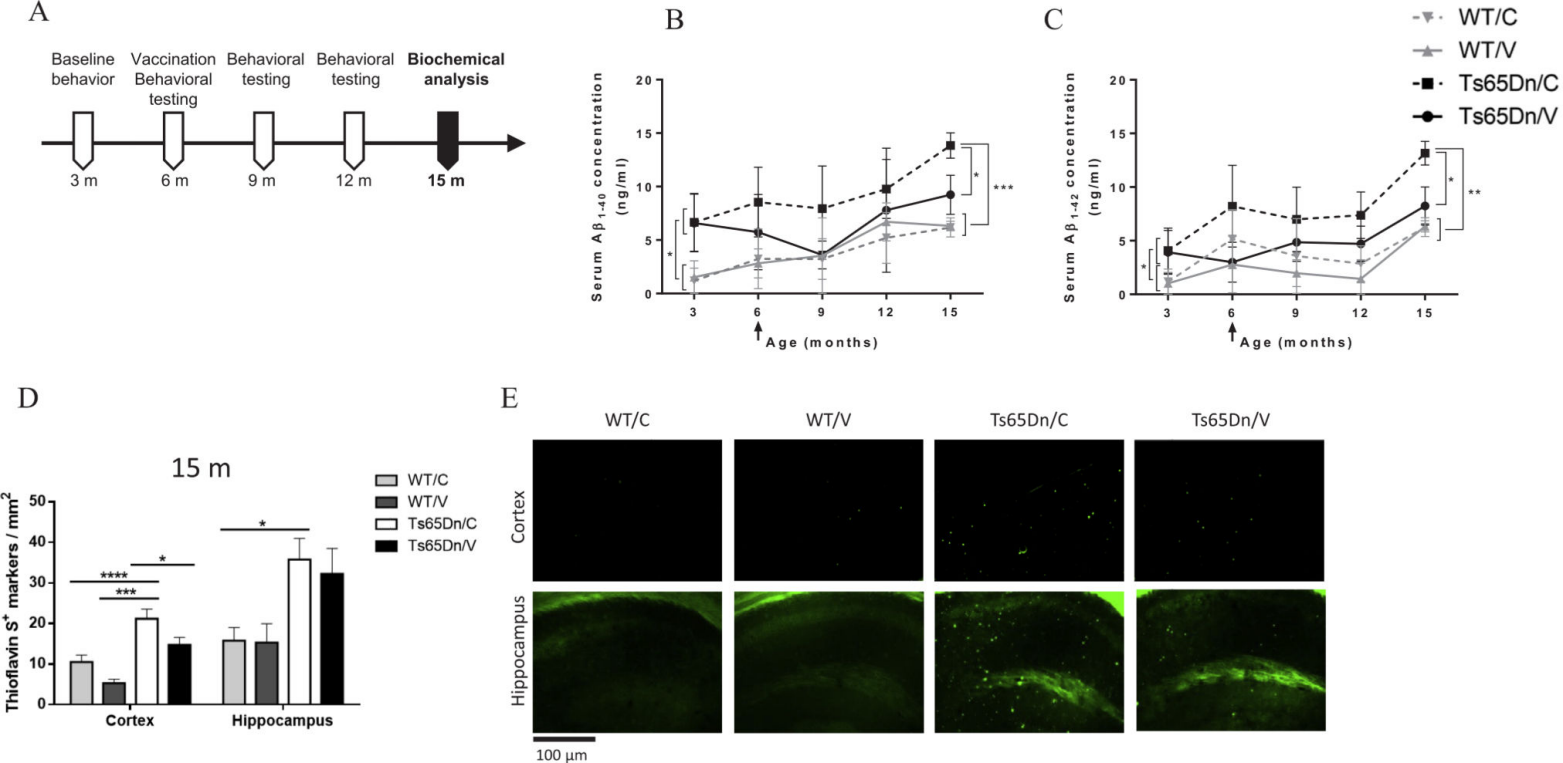
Vaccination with AβCore-S reduces serum Aβ40, Aβ42 levels and alleviate amyloidic burden in the cerebral cortex of 15 m-old Ts65Dn mice. (A) Serum levels of Aβ40, Aβ42 was assessed every 3 months using sELISA. Amyloidic burden was assessed using Thioflavin-S stain at 15 m of age. (B) Reduced Aβ40 levels in the sera of vaccinated Ts65Dn mice, after immunization and at 15 m of age compared with sham-vaccinated Ts65Dn mice (P < 0.05). (C) Reduced Aβ42 levels in the sera of vaccinated Ts65Dn mice, after immunization and at 15 m of age compared with sham-vaccinated Ts65Dn mice (P < 0.05). (D) Vaccinating Ts65Dn mice with AβCore-S resulted in a reduction in ThioS+ markers in their cerebral cortex at 15 m of age, compared with sham-vaccinated Ts65Dn mice (P < 0.05). Sham-vaccinated Ts65Dn mice presented a higher number of ThioS+ markers compared with WT mice in the cerebral cortex (P < 0.001) and hippocampus (P < 0.05). (E) Thioflavin-S stain of the cortex and hippocampus. Two-way ANOVA, Repeated-measures Two-way ANOVA, *P < 0.05, **P < 0.01, ***P < 0.001, ****P < 0.0001.

To assess the efficacy of the AβCore-S vaccine in targeting Aβ_1–40_ and Aβ_1–42_, serum levels of these peptides were measured at base-line (3 m) and every 3 m following immunization (Fig. 4A). Levels of Aβ_1–40_ in the sera of Ts65Dn mice were higher compared to WT mice at 3 m (6.62 ± 3.38 ng/ml and 1.36 ± 1.3 ng/ml, respectively, P < 0.05, Fig. 4B). Similarly, serum levels of Aβ_1–42_ were also higher in these mice at 3 m of age (4 ± 2.04 ng/ml and 1.1 ± 1.1 ng/ml, respectively, P < 0.05, Fig. 4C). Following immunization, serum levels of both Aβ_1–40_ and Aβ_1–42_ were lower in vaccinated Ts65Dn mice, which reached significance level at the age of 15 m compared with sham-vaccinated Ts65Dn mice (9.23 ± 1.82 ng/ml and 13.83 ± 1.18 ng/ml respectively for Aβ_1–40_; 8.23 ± 1.76 ng/ml and 13.15 ± 1.1 ng/ml, respectively for Aβ_1–42_. P < 0.05, Fig. 4B, C). Serum Aβ_1–40_ and Aβ_1–42_ were lower in WT controls compared with sham-treated mice at the age of 15 m (6.24 ± 0.66 ng/ml and 13.83 ± 1.18 ng/ml respectively for Aβ_1–40_, P < 0.001; 6.3 ± 0.69 ng/ml and 13.15 ± 1.1 ng/ml respectively for Aβ_1–42_, P < 0.01, Fig. 4B, C), but not compared to vaccinated Ts65Dn mice (P = 0.77 for Aβ_1–40_, P = 0.83 for Aβ_1–42_ Fig. 4B, C).

Number of Thioflavin-S^+^ markers was assessed in the cerebral cortex and hippocampus of Ts65Dn and WT mice from 15 m old mice. The number of Thioflavin-S^+^ markers was lower in the cortex of vaccinated Ts65Dn mice compared with sham-vaccinated Ts65Dn mice (14.76 ± 9.73 and 21.19 ± 14.22 markers/mm^2^ Respectively, P < 0.05, Fig. 4D, E). No significant differences were observed in the hippocampus (P = 0.99, Fig. 4D).

**Fig. 5.**
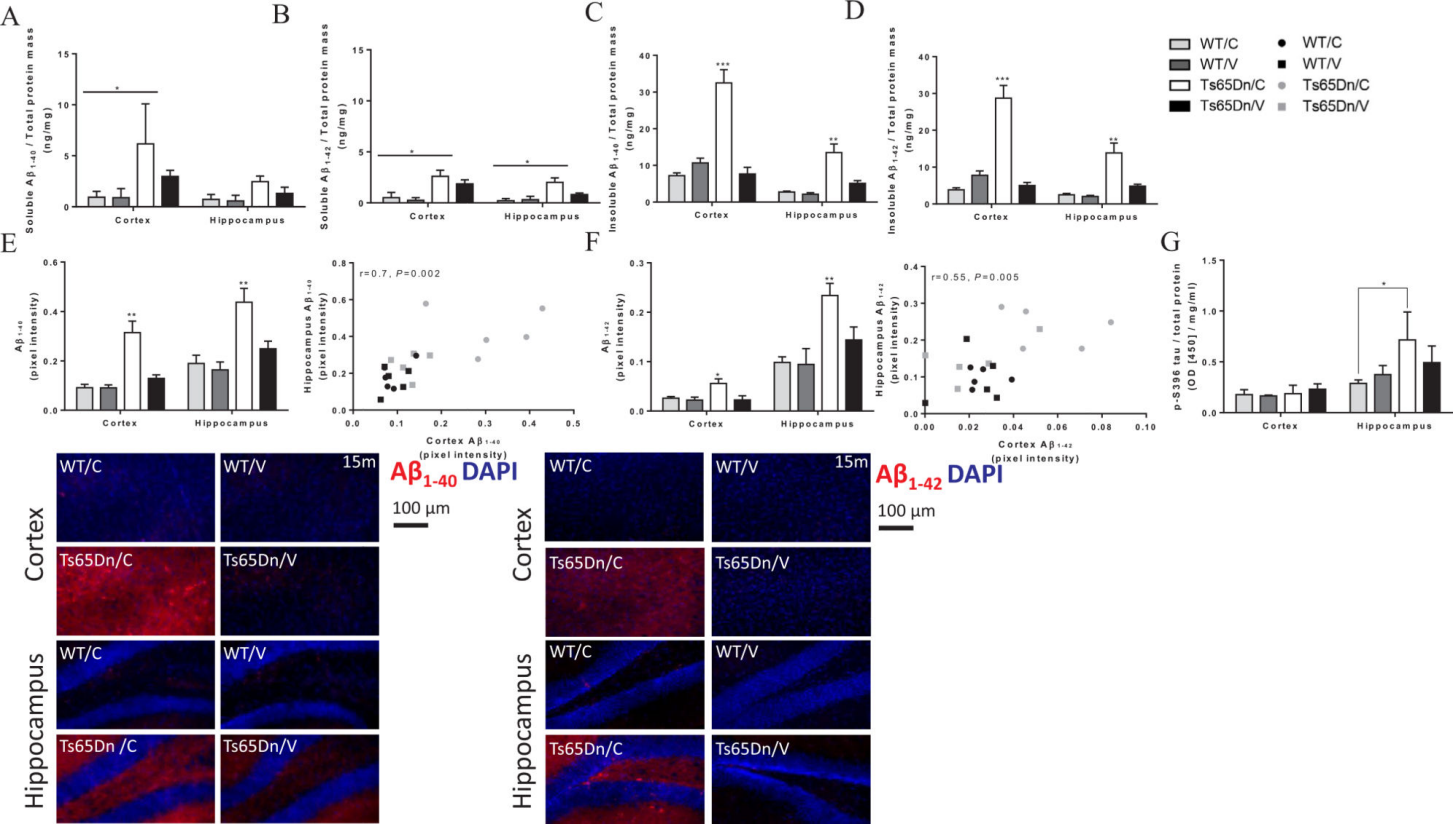
Vaccinated Ts65Dn mice exhibit lower levels of cerebral, soluble and insoluble Aβ40/42 and S396-P-tau at 15 m of age. Tissue levels of S396-P-tau protein, soluble and insoluble Aβ40 and Aβ42 were measured quantitatively using sELISA (A-D, G) and IHC (E-F). (A) Cortical levels of soluble Aβ40 were higher in sham-vaccinated Ts65Dn mice compared with both WT mice (P < 0.05). (B) Cortical and hippocampal levels of soluble Aβ42 were higher in sham-vaccinated Ts65Dn mice compared with both WT mice (P < 0.05). (C) Cortical and hippocampal levels of insoluble Aβ40, were higher in sham-vaccinated Ts65Dn mice compared with vaccinated Ts65Dn and WT mice (P < 0.001, P < 0.01, respectively). (D) Cortical and hippocampal levels of insoluble Aβ42 were higher in sham-vaccinated Ts65Dn mice compared with vaccinated Ts65Dn and WT mice (P < 0.001, P < 0.01, respectively). (E) IHC analysis for Aβ40 reveals higher levels in the cortex and hippocampus of sham-vaccinated Ts65Dn mice compared with vaccinated Ts65Dn and WT mice (P < 0.01, upper-left and bottom panels), with high positive correlation between measurements in the cortex and hippocampus (Pearson’s *r* = 0.7, P < 0.05, upper-right panel). (F) IHC analysis for Aβ42 reveals higher levels in the cortex and hippocampus of sham-vaccinated Ts65Dn mice compared with vaccinated Ts65Dn and WT mice (P < 0.05 for the cortex and P < 0.01 for the hippocampus, upper-left and bottom panels), with medium positive correlation between measurements in the cortex and hippocampus (Pearson’s *r* = 0.55, P < 0.05, upper-right panel). (G) Hippocampal levels of S396-P-tau protein were higher in sham-vaccinated Ts65Dn mice compared with sham-vaccinated WT mice (P < 0.05), Repeated-measures Two-way ANOVA, Pearson’s correlation coefficient, *P < 0.05, **P < 0.01, ***P < 0.001.

Next, cortical and hippocampal levels of Aβ_1–40_ and Aβ_1–42_ were quantified using sELISA as previously described (Illouz et al., 2017). Cortical level of soluble Aβ_1–40_ were higher in sham-vaccinated Ts65Dn mice compared with WT controls (6.13 ± 3.93 ng/ml and 0.93 ± 0.57 ng/ml, respectively, P < 0.05, Fig. 5A). No difference was observed in the levels of soluble Aβ_1–40_ levels in the hippocampus (P = 0.66, Fig. 5A). Vaccinated Ts65Dn mice showed no difference in soluble Aβ_1–40_ compared with WT controls in either the cortex (P = 0.79, Fig. 5A) or hippocampus (P = 0.97, Fig. 5A).

Soluble levels of Aβ_1–42_ were elevated in sham-vaccinated Ts65Dn mice compared with WT mice in both the cortex (2.6 ± 0.59 ng/ml and 0.51 ± 0.51 ng/ml, respectively, P < 0.05 Fig. 5B) and hippocampus (2.01 ± 0.43, 0.2 ± 0.2 ng/ml, respectively, P < 0.05 Fig. 5B). However, there was no difference between vaccinated Ts65Dn mice and their WT controls (P = 0.06 in the cortex, P = 0.94 in the hippocampus, Fig. 5B). Furthermore, levels of insoluble Aβ_1–40_ were lower in the cortex and hippocampus of vaccinated Ts65Dn mice compared with sham-vaccinated Ts65Dn mice (Cortex: 7.66 ± 1.77 ng/ml and 32.48 ± 3.63 ng/ml, respectively P < 0.001; Hippocampus: 5.08 ± 0.78 ng/ml and 13.51 ± 2.3 ng/ml, respectively, P < 0.01, Fig. 5C). Accordingly, tissue levels of insoluble Aβ_1–40_ showed no difference between vaccinated Ts65Dn mice and WT controls (P = 0.81 in the cortex, P = 0.65 in the hippocampus, Fig. 5C). Additionally, vaccinated Ts65Dn mice exhibited reduced levels of insoluble Aβ_1–42_ in the cortex and the hippocampus, compared with sham-vaccinated Ts65Dn mice (Cortex: 4.99 ± 0.86 ng/ml and 28.73 ± 3.46 ng/ml, respectively, P < 0.001, Fig. 5D; Hippocampus: 4.83 ± 0.53 ng/ml and 13.83 ± 2.71 ng/ml, Respectively, P < 0.01, Fig. 5D). No difference was observed in either cortical or hippocampal levels of insoluble Aβ_1–42_ between vaccinated Ts65Dn mice and controls (P = 0.65 in the cortex, P = 0.64 in the hippocampus, Fig. 5D).

Immunohistochemical staining for Aβ_1–40_ revealed lower pixel intensity (PI) in the cortex and hippocampus of vaccinated Ts65Dn mice compared with sham-vaccinated Ts65Dn mice (0.12 ± 0.01 and 0.31 ± 0.04 PI, in the cortex, 0.24 ± 0.03 and 0.43 ± 0.05 PI, in the hippocampus, respectively, P < 0.01, Fig. 5E). Additionally, a strong correlation between cortical and hippocampal Aβ_1–40_ expression levels was observed (*r* = 0.7, P < 0.01, Fig. 5E, right panel). Similarly, we found reduced Aβ_1–42_ signal in the cortex and hippocampus of vaccinated Ts65Dn mice compared with sham-vaccinated Ts65Dn mice (0.02 ± 0.009 and 0.05 ± 0.009 PI, in the cortex, respectively, P < 0.05, 0.14 ± 0.02 and 0.23 ± 0.02 PI, in the hippocampus, respectively, P < 0.01, Fig. 5F). A moderate correlation between cortical and hippocampal signal intensity of Aβ_42_ was found (*r* = 0.55, P < 0.01, Fig. 5F).

To further investigate the impact of the AβCore-S vaccine on AD-like neuropathology found in DS patient, we measured levels of phosphorylated tau (S396) in the cortex. Hippocampal but not cortical pS396-tau levels of sham-vaccinated Ts65Dn mice were elevated compared to WT controls (0.71 ± 0.27 and 0.28 ± 0.03 OD, respectively, P < 0.05, Fig. 5G). This is in line with previous reports of *DYRK1A* overexpression in hippocampal CA1 pyramidal neurons of DS patients (Wegiel et al., 2011). Vaccinated Ts65Dn mice did not show difference in cortical or hippocampal levels of p-S396-tau, compared to WT mice (P = 0.98 in the cortex, P = 0.91 in the hippocampus, Fig. 5G).

### 3.6. Vaccination with AβCore-S reduces cortical neurodegeneration in Ts65Dn mice

**Fig. 6.**
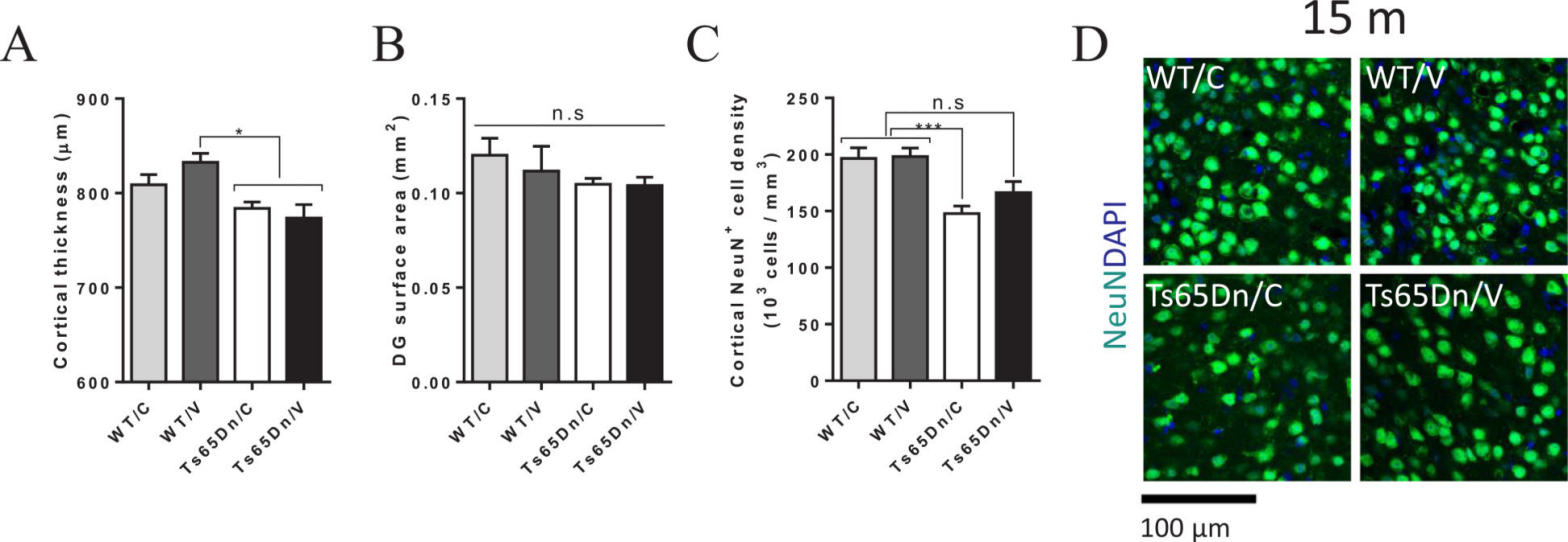
Vaccination with AβCore-S reduces cortical neurodegeneration in 15 m-old Ts65Dn mice. Effects on neurodegeneration were assessed by measuring cortical thickness, dentate gyrus surface area and cortical neuronal density. (A) Ts65Dn exhibited reduced cortical thickness, regardless of treatment (P < 0.05), however (B) the surface area of the dentated gyrus did not differ from WT mice (P = 0.41). Importantly, (C, D) NeuN^+^ cell density in the cortex of sham-treated Ts65Dn mice was lower than measured in WT mice (P < 0.001), whereas vaccinated Ts65Dn mice resembled healthy controls (P = 0.063). Two-way ANOVA, Repeated-measures Two-way ANOVA, *P < 0.05, ***P < 0.001.

Along with neurodevelopmental alterations during embryonic stages, DS individuals suffer from early AD-associated neurodegeneration of the hippocampus, amygdala and cortex (Kesslak et al., 1994; Krasuski et al., 2002; Teipel et al., 2004; Teipel et al., 2003). In Ts65Dn mice, APP overexpression, together with associated neuroinflammation and oxidative stress, has been implicated in the degeneration of cholinergic and noradrenergic neurons (Millan Sanchez et al., 2012). We next inquired whether vaccination-derived reduction in Aβ levels is associated with restricted neurodegeneration in immunized Ts65Dn mice. Cortical thickness of vaccinated and sham-treated Ts65Dn mice was reduced compared with vaccinated WT controls (776.6 ± 9.6 and 832.6 ± 9.48 μm, respectively, P < 0.05, Fig. 6A), independently of treatment (P = 0.98, Fig. 6A). Additionally, dentate gyrus (DG) area did not differ between strains or treatments (P = 0.59, Fig. 6B). Next, neuronal density was assessed using unbiased stereological analysis of NeuN^+^ neurons in the retrosplenial cortex, as Ts65Dn mice were identified for cholinergic circuitry deficit in the retrosplenial cortex (Chen et al., 2009). Importantly, the neuronal density of sham-treated Ts65Dn mice was lower compared to sham-treated and vaccinated WT controls (147.8 ± 6.59, 196.5 ± 9.39 and 198.3 ± 7.33 × 10^3^ cells/mm^3^, respectively, P < 0.05, Fig. 6C, D). The neuronal density among vaccinated Ts65Dn mice was also reduced, however this effect did not reach significance level compared with WT controls of both groups (166 ± 6.559, 196.5 ± 9.39 and 198.3 ± 7.33 × 10^3^ cells/mm^3^, respectively, P = 0.06, Fig. 6C, D). These data indicate that the relief in amyloidic burden in vaccinated Ts65Dn mice has a mild positive effect on neuronal integrity in the retrosplential cortex.

### 3.7. Vaccination with AβCore-S alters microglia protein expression profile and promotes homeostatic microglial phenotype

Microglia have recently been implemented in neurodegenerative and other CNS-related pathologies, as activation of these cells under pathological states and loss of their homeostatic function may contribute to neuronal and synapses loss by promoting harmful inflammation in the brain parenchyma (Bisht et al., 2016; Colonna and Butovsky, 2017; Keren-Shaul et al., 2017; Yin et al., 2017). It has also been reported that DS individuals, as well as mouse models of DS, exhibit a unique microglial gene expression signature that is distinct from familial and sporadic AD (Wilcock et al., 2015; Xue and Streit, 2011). We characterized the phenotype of microglial cells by measuring the expression of pan-microglial, homeostatic and reactive microglia markers. To examine the broad population of microglial cells, we used the Ionized calcium binding adaptor molecule 1 (Iba1), a member of the calcium-binding group of proteins (Korzhevskii and Kirik, 2016), uniformly distributed in the cytoplasm and processes of ramified microglia. Iba1 takes part in reorganizing the cytoskeleton and altering the configuration of the plasmalemma, processes that occur during phagocytosis (Kawai et al., 2005; Kohler, 2007; Korzhevskii and Kirik, 2016). For homeostatic microglial cells we used the 4D4 and P2RY12 markers. 4D4, a newly discovered microglia-specific marker of unknown function, expressed specifically at the extremity of the cells’ ramified processes (Bisht et al., 2016). Purinergic receptor P2RY12 (G-protein coupled, 12), another homeostatic microglial marker, is downregulated under ischemic and neurodegenerative conditions (Bisht et al., 2016; Keren-Shaul et al., 2017; Lou et al., 2016). Finally, we used CD68 and Clec7a as markers for reactive microglial cells. CD68, a transmembrane protein present in monocytes and tissue macrophages, belongs to the lysosome-associated membrane protein family and is indicative of phagocytic activity (Korzhevskii and Kirik, 2016; Walker and Lue, 2015). Clec7a (Dectin-1) is a C-type lectin receptor, that is associated with plaque-related microglia (Keren-Shaul et al., 2017; Osorio and Reis e Sousa, 2011).

**Fig. 7.**
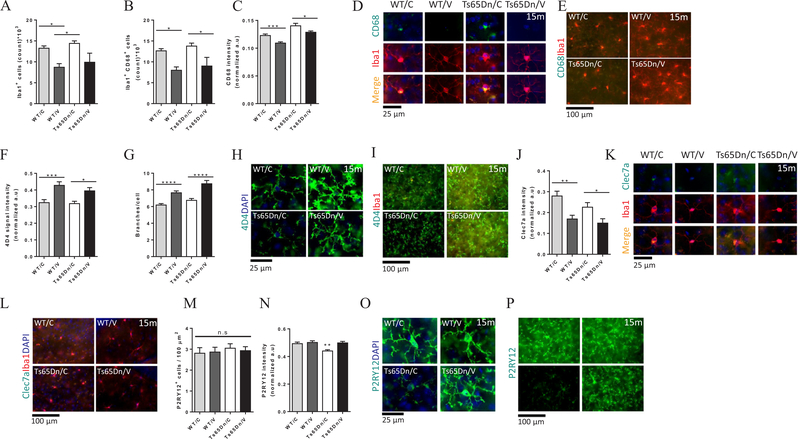
Vaccination with AβCore-S alters microglia protein expression profile and promotes homeostatic microglial phenotype. Microglial protein expression phenotype was conducted by IHC targeting pan (Iba1, 4D4), reactive (CD68, Clec7a) and homeostatic (P2RY12) microglia at 15 m of age. (A) Iba1+ cell count, showing gliosis in the hippocampi sham-vaccinated Ts65Dn mice compared with vaccinated WT mice, (B) higher number of Iba1+/CD68+ cells in the hippocampi of sham-treated Ts65Dn and WT mice compared with both vaccinated transgenic and WT mice, (C) CD68 fluorescent signal is higher in sham-treated Ts65Dn and WT mice, compared with vaccinated mice of both strains (D, E) Exemplars of Iba1/CD68 IHC representing higher CD68 expression in sham-treated mice, magnification of ×63, ×40 respectively. (F) Expression of 4D4 is elevated in vaccinated Ts65Dn and WT mice, compared with sham-treated mice. (G) Number of branches/cell is higher in vaccinated Ts65Dn and WT mice compared with sham-treated mice. (H, I) Exemplars of 4D4 IHC representing higher 4D4 expression in vaccinated mice compared with sham-treated mice, magnification of ×63, ×40 respectively. (J) Expression of Clec7a on microglial cell is higher in sham-treated Ts65Dn and WT mice, compared with vaccinated mice (K, L) Exemplars of Clec7a IHC, magnification of ×63, ×40 respectively. (M) Number of P2RY12+ cells in the hippocampus does not change between vaccinated and sham-treated Ts65Dn and WT mice. (N) Sham-treated Ts65Dn mice exhibit lowered expression of the homeostatic marker P2RY12 in hippocampal microglial cell, compared with vaccinated Ts65Dn mice and healthy controls. (O, P) Exemplars of P2RY12 IHC representing lowered expression in sham-treated Ts65Dn mice, compared with all other groups, magnification of ×63, ×40 respectively. Repeated-measures Two-way ANOVA, *P < 0.05, **P < 0.01, ***P < 0.001, ****P < 0.0001.

Ts65Dn mice did not exhibit higher numbers of hippocampal Iba1^+^ cells when compared with WT mice (14.42 × 10^3^ ± 0.58 × 10^3^ and 13.25 × 10^3^ ± 0.55 × 10^3^ respectively, P > 0.05, Fig. 7A). Compared with sham-vaccinated WT mice, vaccinated WT mice exhibited reduced levels of Iba1^+^ cells in the hippocampus (13.25 × 10^3^ ± 0.55 × 10^3^ and 8.71 × 10^3^ ± 0.86 × 10^3^ respectively, P < 0.05, Fig. 7A). Vaccinated Ts65Dn mice exhibited nonsignificant reduction in the numbers of Iba1^+^ cells within the hippocampus compared with sham-treated Ts65Dn mice (9.9 × 10^3^ ± 0.21 × 10^3^ and 14.42 × 10^3^ ± 0.58 × 10^3^ respectively, P = 0.07, Fig. 7A). This effect implies that the AβCore-S vaccine reduces microgliosis in both WT and Ts65Dn aging brains. Importantly, we found that the number of Iba1^+^CD68^+^ microglial cells in the hippocampus of vaccinated Ts65Dn and WT mice (9.01 × 10^3^ ± 0.2 × 10^3^ and 7.99 × 10^3^ ± 0.8 × 10^3^, respectively, Fig. 7B, D and E) was lower compared with sham-vaccinated Ts65Dn and WT mice (13.77 × 10^3^ ± 0.73 × 10^3^ and 12.64 × 10^3^ ± 0.52 × 10^3^ respectively, P < 0.05, Fig. 7B, D and E). In addition, CD68 expression was reduced in vaccinated Ts65Dn and WT mice (0.12 ± 0.002 and 0.1 ± 0.002 respectively, Fig. 7C-E), compared to sham-vaccinated Ts65Dn and WT mice (0.14 ± 0.004 and 0.12 ± 0.002 respectively, Fig. 7C-E, P < 0.05 for Ts65Dn, P < 0.001 for WT). These results suggested that the AβCore-S vaccine shifts the polarity of microglial cells toward a homeostatic rather than pathologic phenotype, regardless of DS-related pathology. To clarify this, we next assessed the morphology of microglial cells using the homeostatic microglia marker 4D4 (Bisht et al., 2016), expressed intensively at the extremity of microglial processes. Vaccinated Ts65Dn and WT mice exhibit elevated expression of 4D4 (0.39 ± 0.01 and 0.42 ± 0.02 respectively, Fig. 7F, H and I) compared with sham-vaccinated Ts65Dn and WT mice (0.31 ± 0.01 and 0.32 ± 0.01 respectively, Fig. 7F, H and I, P < 0.05 for Ts65Dn, P < 0.001 for WT). Interestingly, microglial cells in vaccinated mice of both strains exhibited a higher number of branches per cell (Ts65Dn: 8.73 ± 0.39; WT: 7.65 ± 0.23, Fig. 7G-I) compared with unvaccinated mice (Ts65Dn: 6.47 ± 0.22; WT: 6.18 ± 0.17, P < 0.0001. Fig. 7G-I). Reduced ramification in sham-vaccinated mice is consistent with higher CD68 expression and implies elevated age-related microglial activation in these mice. To confirm this, we also assessed levels of Clec7a, a marker recently reported to be upregulated in Aβ plaque-associated reactive microglia (Keren-Shaul et al., 2017). Clec7a expression intensity was reduced in vaccinated Ts65Dn and WT mice (0.14 ± 0.02 and 0.16 ± 0.01 respectively, Fig. 7J-L) compared to sham-vaccinated Ts65Dn and WT mice (0.22 ± 0.02 and 0.27 ± 0.02, P < 0.05, P < 0.01, respectively, Fig. 7J-L). Finally, expression level of P2RY12, a marker of homeostatic microglia, were assessed, as this marker severely downregulated in reactive microglia found in the brains of AD mouse models (Keren-Shaul et al., 2017; Moore et al., 2015). While no difference was noted in the number of P2RY12^+^ microglial cells between groups (P = 0.33, Fig. 7M, O and P), P2RY12 expression levels were lower in sham-vaccinated Ts65Dn mice, compared with vaccinated Ts65Dn mice (0.43 ± 0.009 and 0.49 ± 0.01, respectively, P < 0.01 Fig. 7N-P). In contrast, no difference in P2RY12 expression level was noted between sham and vaccinated WT mice (0.5 ± 0.01, 0.49 ± 0.01, respectively, P = 0.95, Fig. 7N-P). The concomitant up-regulation of the reactive markers CD68 and Clec7a and the down regulation of P2RY12, along with reduced ramification in sham-treated Ts65Dn mice suggests that the AβCore-S vaccine reduces microglial activation and promotes microglial homeostasis in the brain parenchyma.

### 3.8. Vaccination with AβCore-S reduces S100β+ and C3^+^ astrocytes reactivity in the cortex and hippocampus of Ts65Dn mice

**Fig. 8.**
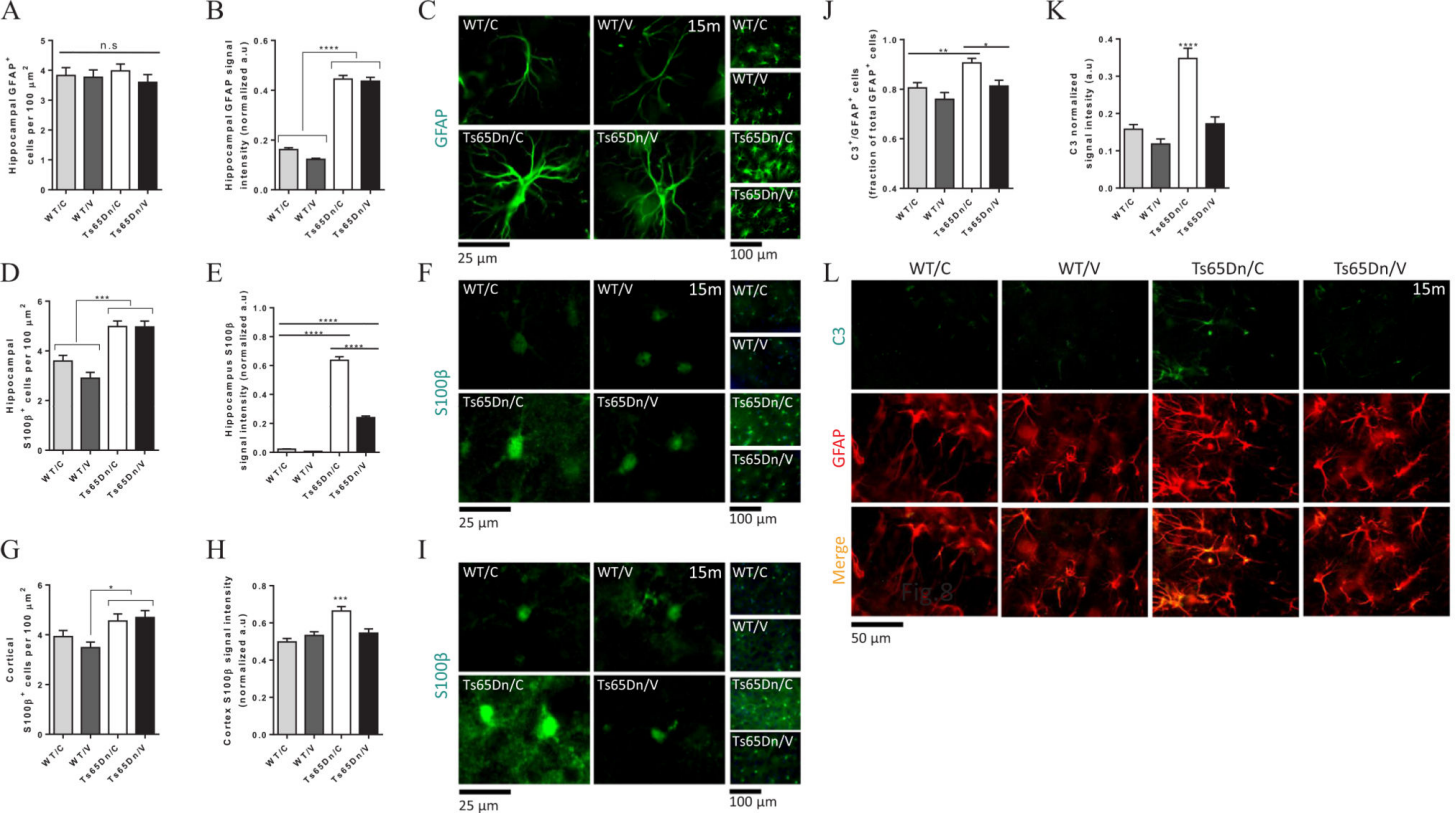
Vaccination with AβCore-S reduces S100β+ and C3-expressing astrocytes reactivity in Ts65Dn mice. GFAP+, S100β+ and C3-reactive astrocytes were measured using stereology at 15 m of age. (A) Hippocampal GFAP+ cell counts, showing no difference between Ts65Dn and WT mice. (B) GFAP intensity is higher in vaccinated and sham-treated Ts65Dn mice compared with healthy controls. (C) Representative attaining of GFAP+ cells showing higher expression levels in the hippocampus of Ts65Dn mice, compared with controls, magnification of ×63 (left panels), ×40 (right vertical panels) (D) Hippocampal S100β+ cells indicate astrogliosis in vaccinated and sham-treated Ts65Dn mice compared with WT controls. (E) Elevated hippocampal S100β intensity signal in sham-treated Ts65Dn mice compared with vaccinated Ts65Dn mice and healthy controls. (F) Representative images of S100β+ cells showing higher expression in the hippocampus of sham-treated Ts65Dn mice, compared with all other groups, magnification of ×63 (left panels), ×40 (right vertical panels) (G) Cortical S100β+ cells indicate astrogliosis in vaccinated and sham-treated Ts65Dn mice compared with vaccinated but not sham-treated WT mice (H) Elevated cortical S100β intensity in sham-treated Ts65Dn mice compared with vaccinated Ts65Dn mice and healthy controls (I) Representative images of S100β+ cells indicating higher expression in the cortex of sham-treated Ts65Dn mice, compared with all other groups, magnification of ×63 (left panels), ×40 (right vertical panels). (J) Sham-treated Ts65Dn mice exhibited higher fraction of C3-expressing GFAP + hippocampal astrocytes compared with vaccinated (P < 0.05) and healthy controls (P < 0.01). Additionally, (K) higher C3 signal intensity was found in these mice compared with all other groups (P < 0.001). (L) Exemplars of C3-expressing GFAP + hippocampal astrocytes. Repeated-measures Two-way ANOVA, *P < 0.05, **P < 0.01, ***P < 0.001, ****P < 0.0001.

Astrocytes and microglial reactivity, both contribute to the pathogenesis of AD (Garwood et al., 2017; Guenette, 2003; Liddelow et al., 2017; Rodriguez-Arellano et al., 2016; Verkhratsky et al., 2010) and DS (Chen et al., 2014; Lockrow et al., 2012; Lu et al., 2011; Sebastia et al., 2004). Astrocytes are involved in Aβ clearance and promote tissue homeostasis (Garwood et al., 2017; Guenette, 2003; Liddelow et al., 2017; Sollvander et al., 2016; Wyss-Coray et al., 2003). However, oligomeric Aβ induces the secretion of inflammatory molecules from astrocytes, such as IL-1β, iNOS and in turn, overproduction of NO (White et al., 2005). Individuals with DS, exhibit chronic inflammation with increased astrocytic activation, expressed as elevation in S100β and GFAP expression, also found in Ts65Dn mice (Lockrow et al., 2012; Lu et al., 2011), coupled with IL-1β and TNF-α cytokine release (Lockrow et al., 2012). S100β overexpression correlates with the pattern of regional neuropathology and neuritic plaques in AD (Hu et al., 1996; Lu et al., 2011). Importantly, the S100B gene is located on the triplicated Hsa21, and thus are being overexpressed in DS individuals. However, it is not triplicated in the Ts65Dn model. Additionally, a recent study has shown that neurodegeneration-related reactive astrocytes (A1 phenotype), upregulate the expression of complement component 3 (C3) (Liddelow et al., 2017). To examine the effect of the AβCore-S vaccine on astrocytic phenotype in Ts65Dn mice, we conducted stereological analysis of GFAP^+^, S100β^+^ and C3^+^ cells in the cortex and the hippocampus. The number of GFAP^+^ cells in the hippocampus did not differ between vaccinated and sham-treated Ts65Dn mice (3.59 ± 0.26 and 3.98 ± 0.23 cells/100 μm^2^, respectively, P = 0.68, Fig. 8A) and resembled the number of cells found in vaccinated and sham-treated WT mice (3.77 ± 0.24 and 3.82 ± 0.26 cells/100 μm^2^, respectively, P = 0.74, Fig. 8A). However, hippocampal GFAP intensity levels was higher in vaccinated and sham-treated Ts65Dn mice (0.43 ± 0.01 and 0.44 ± 0.01, respectively, Fig. 8B, C) compared with vaccinated and sham-treated WT mice (0.12 ± 0.0043 and 0.16 ± 0.0067, respectively, P < 0.0001, Fig. 8B, C). Consistently with previous report (Chen et al., 2014), this finding suggests that while astrocytes in Ts65Dn mice are more activated than their WT counterparts, the vaccination did not altered the basal GFAP expression in both strains.

Quantifying S100β^+^ cells, we found higher number of S100β^+^ cells in the hippocampus of vaccinated and sham-treated Ts65Dn mice (4.96 ± 0.23 and 4.98 ± 0.21 cells/100 μm^2^, respectively, Fig. 8D) compared with vaccinated and sham-treated WT mice (2.9 ± 0.23 and 3.59 ± 0.22 cells/100 μm^2^, P < 0.001). This result suggests the astrogliosis is taking place in Ts65Dn hippocampi. Surprisingly, vaccinating Ts65Dn mice significantly reduced S100β expression compared with sham-treated Ts65Dn mice (0.24 ± 0.01 and 0.63 ± 0.02 a.u, respectively, P < 0.0001, Fig. 8E, F). Overexpression of S100β, previously reported in this strain (Lu et al., 2011), as well as in DS individuals (Griffin et al., 1989; Hu et al., 1996), is thought to promote tissue damage via inflammation. A reduction of this marker in vaccinated mice is therefore an evidence of tissue homeostasis.

Similar effects were observed in the cortex, as vaccinated and sham-treated Ts65Dn exhibited increased numbers of S100β^+^ cells (4.69 ± 0.27 and 4.55 ± 0.28 cells/100 μm^2^, respectively, Fig. 8G) compared with vaccinated but not sham-treated WT mice (3.48 ± 0.22 and 3.92 ± 0.24, P < 0.05, P = 0.23, respectively, Fig. 8G). Sham-treated Ts65Dn mice exhibited higher S100β intensity in the cortex compared with vaccinated Ts65Sn mice (0.66 ± 0.02 and 0.54 ± 0.02, respectively, P < 0.001 Fig. 8H, I) and healthy controls. Additionally, vaccinated Ts65Dn mice exhibited similar S100β intensity as vaccinated and sham-treated controls (0.54 ± 0.02 and 0.53 ± 0.01, 0.49 ± 0.01, P = 0.67, Fig. 8H, I).

We next examined the expression of complement component 3 (C3), a marker recently reported to be expressed on A1-neurodegeneration-related reactive astrocytes. Sham-treated Ts65Dn mice exhibit a higher number of C3^+^ astrocytes within the GFAP^+^ astrocytes population in the hippocampus, compared with vaccinated Ts65Dn mice (0.9 ± 0.1 and 0.81 ± 0.02, respectively, P < 0.05, Fig. 8J), and healthy controls (0.8 ± 0.02 and 0.76 ± 0.02, respectively, P < 0.01, Fig. 8J). Importantly, elevated fraction of C3 expressing cells was accompanied by higher C3 expression in sham-treated Ts65Dn mice compared with vaccinated mice (0.34 ± 0.02 and 0.17 ± 0.01 a.u., respectively, P < 0.0001 Fig. 8K, L), and WT controls (0.15 ± 0.01 and 0.11 ± 0.01 a.u., respectively, P < 0.0001 Fig. 8K, L).

These results suggest that while not affecting the number of GFAP^+^ or S100β^+^ astrocytes, the AβCore-S vaccine reduced the expression levels of both S100β^+^ and C3^+^ neurodegeneration-related reactive astrocytes.

## 4. Discussion

In the current study, Ts65Dn mouse model of human Down syndrome were immunized against murine Aβ_1–11_ and assessed whether it exerts a beneficial effect on various aspects of cognition, i.e. exploratory behavior, anxiety, long-term spatial memory and short-term memory. Additionally, we investigated the therapeutic effects of the AβCore-S vaccine in reducing neuropathological hallmarks of Alzheimer’s disease, which contributes to the pathogenesis of Down syndrome. Among these, are elevation in cerebral soluble-Aβ levels, accumulation of insoluble Aβ, tauopathy, microglial and astroglial activation.

The Ts65Dn strain provides a powerful tool in modeling the detrimental effect of Aβ overexpression found in human DS patients. This is due to the context in which Aβ being overexpressed and accumulated. First, the chromosomal abnormality originates in the early stages of embryonic development leading to elevated levels of APP and Aβ in the brain throughout life, rather than in late stages as found in AD. In addition, APP is overexpressed in the Ts65Dn model along with a milieu of Hsa21-located genes, providing a unique transcriptome and proteome in which insoluble Aβ accumulation initiates in early adulthood. In contrast to most AD mouse models, Ts65Dn mice encompass overexpression of the endogenous murine APP rather than an exogenous human Aβ.

Ts65Dn mice exhibit a complex behavioral phenotype which affects the assessment of their cognitive behavior. Indeed, the observed hyperactivity affected performance of the mice in the Barnes maze task. Hyper-movement can interfere with spatial learning, as the animal predominantly adopts cognitive but non-spatial strategies such as *random search*. This phenotype, however, is markedly different from the impairment of Ts65Dn mice in water-based tasks.

Ts65Dn mice perform normally in open environments, as they spend normal fractions of time in the center and periphery of the open field arena, suggesting their exploratory behavior is intact. However, we found an age-consistent elevation in exploring the anxiogenic zones of the elevated zero maze, evidencing higher anxiety threshold in these mice. This finding can directly influence their performance in the Barnes maze, in which the animal is motivated to find a hiding chamber by being exposed to a moderate-stressful environment.

Latency to reach the target hole in the Barnes maze was similar in Ts65Dn and WT mice at baseline measurement as well as post-immunization at 9 m of age, suggesting absence of anxiety interference or abnormality in motivation. Accordingly, walking speed of Ts65Dn mice was consistently higher and their path efficiency was lower than of WT. To address this dissonance, we tested mice in the probe test and in a following reversal task. These paradigms demand higher cognitive resources, thus most of the treatment effects emerged within these tests. Second, a deeper understanding of the animals’ cognitive state can be obtained by analyzing their searching strategy more carefully. To do so, we applied the BUNS classifier, providing us with further insights regarding their performance in the Barnes maze. Overall, the BUNS results indicate that the equal latencies we found earlier did not reflect similar learning capacities of Ts65Dn and WT mice, but rather a compensation strategy utilized by Ts65Dn mice. That is, these mice used lower spatial strategies (i.e. *serial search*) to enhance their chance of finding the target in the unlearned environment.

The AβCoreS vaccine, induces the expression of an Aβ_1–11_ peptide fused to the Hepatitis-B surface antigen (HBsAg) (Olkhanud et al., 2012). The construct also contains the Hepatitis-B capsid antigen (HBcAg) to provide a T helper response that promotes antibody production by B-lymphocytes. DNA vaccines allow controlling antigen-encoding genes by strong mammalian promoters on the plasmid backbone (Khan, 2013). They induce antigen presentation in the context of both major histocompatibility complex-1 (MHC-I) and MHC-2, produce a wider range of immune responses and a long-term persistent of immunogenicity, in comparison with conventional protein-based compounds. Additionally, DNA vaccine are inexpensive, more stable, safer and easy to use (Khan, 2013).

Unlike most familial AD mouse models, Ts65Dn do not develop plaque pathology, but rather overexpress soluble oligomers or small inclusion of insoluble proteins in β-sheet conformation (Lomoio et al., 2009). Importantly, we showed that AβCore-S-induced antibodies can bind Aβ neurotoxic oligomers. Immunization with AβCore-S resulted in a transient, high-titer elevation of serum anti-Aβ IgG and IgM. Antibodies were detectable up to 6 m after vaccination. Importantly, we found that vaccinated WT mice produced higher level of IgG than Ts65Dn, that also decayed over a longer period. In opposite, we found that Ts65Dn mice produced higher levels of IgM than vaccinated control. Lower levels and faster declining of IgG levels together with higher presence of IgM in Ts65Dn mice, may reflect an immune deficiency in antibody production, specifically in class switch mechanism, however this hypothesis need to be further examined. Importantly, we found age-dependent elevation of naturally occurring IgM antibodies (Dodel et al., 2011) in all experimental groups, in a treatment-independent manner. The Aβ-CoreS vaccine targets the endogenous murine Aβ peptide, which is being expressed by WT as well as Ts65Dn mice, and is likely to accumulate with age. Such age-dependent elevation might explain the production of natural autoantibody.

While both Ts65Dn and WT mice produced equivalent levels of anti-Aβ specific IgG1, IgG2a and IgG2b isotypes, WT mice also exhibited increased IgG3 levels. Importantly, IgG1, IgG2b and IgG3 facilitate FcγRn cascade and IgG2b also facilitates FcγRIV cascade.

Interestingly, a different pattern was found for anti-HBsAg antibody production. HBsAg, which serves to prime the immune response, elicited a longer response across all experimental groups, presumably due to its larger antigen and higher immunogenicity compared with endogenous murine Aβ. Importantly, HBsAg-specific IgG and IgM levels did not differ drastically between WT and Ts65Dn, compared with specific anti-Aβ antibodies, suggesting the differential immune response might be associated with different Aβ levels found in WT and Ts65Dn mice, rather than a deficiency in class switch mechanism.

We found that vaccinating Ts65Dn mice with the Aβ-CoreS vaccine rescues specific aspects of behavior and cognition, previously reported to be impaired in these mice. Short-term memory, dramatically impaired in Ts65Dn mice, was spared post immunization, as vaccinated Ts65Dn mice performed similarly to WT controls in the T-maze and Novel-object recognition tasks. Sham-treated Ts65Dn mice showed a lower alternation rate in the T-maze, reflecting reduced short-term memory capacity to encode their last choice. Supporting this finding, sham-treated mice also showed no preference for the novel object in the novel object recognition test. In contrast, immunized Ts65Dn mice performed similarly to healthy controls.

Spatial long-term memory deficit was also improved post-immunization, as sham-treated mice exhibited higher reference and working memory errors and a vast usage of non-spatial searching strategies in the Barnes maze. Vaccinated Ts65Dn mice navigated the environment more efficiently than sham-treated mice, resulting in a stronger memory formation of the target location. This is reflected in a Gaussian-like distribution of hole entries in the probe test of the Barnes maze, centered around the target hole. By comparison, sham-treated mice visit the Barnes maze holes in a uniform manner, reflecting a deficit in hippocampus-dependent long-term memory formation at 9 m of age. Interestingly, this is an age-dependent effect, as naïve 3 m-old Ts65Dn mice exhibit no such deficit. Finally, we found that motor hyperactivity, characterizing Ts65Dn mice, was reduced after immunization.

In association with these behavioral and cognitive effects, we found higher clearance of serum and cerebral Aβ levels, lower hyperphosphorylation of tau protein, reduced neurodegeneration and lower inflammatory phenotype presented by microglial and astroglial cells. Soluble Aβ species, reflecting extracellular oligomers, were reduced in vaccinated Ts65Dn mice to similar levels found in WT mice. Previous reports, suggested that large extracellular aggregates are not essential for neurodegeneration-induced cognitive decline, but rather small Aβ neurotoxic oligomers are held responsible (Gandy et al., 2010; Petersen et al., 2013; Sengupta et al., 2016). Indeed, the Ts65Dn mice lack plaque pathology, but exhibit elevated levels of soluble Aβ and small insoluble inclusions. The main histological characteristic of Aβ in Ts65Dn mice is diffused expression, probably representing the soluble fraction of Aβ, along with small-sized ThioflavinS^+^-reactive foci, reflecting extracellular inclusions. In association with Aβ clearance, a reduction in disease-related cortical neurodegeneration was also evident in vaccinated Ts65Dn mice. Targeting inflammatory activity by Minocycline administration inhibits microglia activation, prevents neuronal loss and improves working and reference memory in Ts65Dn mice (Hunter et al., 2004). Since vaccination-induced Aβ clearance dampens inflammatory markers in microglia and astrocytes, this is a potential pathway that contributes to neuronal survival in vaccinated Ts65Dn mice. Moreover, targeting oxidative stress by antioxidants such as vitamin E, also increases cell density in the DG and reduces cholinergic pathology found in this strain (Lockrow et al., 2009). As the AβCore-S vaccine targets oligomeric Aβ species that promotes oxidative stress, this is also a possible pathway by which neuronal protection was obtained.

It is well established that the pathogenesis of AD, as well as of other neurodegenerative diseases, is tightly associated with microglial phenotype (Colonna and Butovsky, 2017; Jay et al., 2015; Keren-Shaul et al., 2017; Yin et al., 2017). Phagocytosis of Aβ-deposits is largely done by reactive microglial cells, in a process that is facilitated by antigen–antibody binding. On the other hand, hyper-activation of these cells may promote inflammatory environment in the brain parenchyma by secretion of pro-inflammatory cytokines, loss of homeostatic functions, recruitment of monocyte-derived macrophages and infiltration of cytotoxic T lymphocytes, all leading towards neurodegeneration.

Our results indicate that transient vaccination against Aβ in Ts65Dn mice facilitates Aβ clearance, reduces microgliosis in the hippocampus, and helps restore microglial homeostatic features. That is, reduction of microglial hyperactivation, enhanced branching of microglia cells, and elevation of homeostatic markers. It is important to note that since the Ts65Dn strain lacks the presence of large Aβ plaques, which characterize AD models, the microglial phenotype in this strain does not resemble other AD mouse models. First, the morphology of microglia in Ts65Dn mice is characterized by small-soma with ramified processes, rather than amoeboid, as can be seen in fully activated microglia (Caldeira et al., 2014). Second, we did not identify strain-related microgliosis or overexpression of pathologic markers, but rather a treatment-related alteration in cell number and ramification. Importantly, we did find strain and treatment-related restoration of microglial homeostatic phenotype in treated Ts65Dn mice. Despite the differences in Aβ pathology and microglial phenotype, vaccinating 3xtg-AD mice, a plaque-expressing mouse model of AD, against Aβ yielded a reduction in amyloidic load, along with a reduced astrogliosis and microglial activation (Movsesyan et al., 2008). This effect might be related to lower amounts of oligomeric Aβ post immunization, as in the case of vaccinated Ts65Dn mice. Due to the differences in Aβ pathology in DS, deeper understanding of microglial alterations post immunization in Ts65Dn mice, require a comprehensive transcriptomic analysis. The observation that the same vaccine against Aβ_1–11_ can reduce the levels of Aβ in both an AD model which exhibits plaques formation, as well as a DS model which exhibits diffused Aβ expression, is important with respect to its efficacy in different preclinical AD and DS models, as well as in clinical manifestations of Aβ-related pathology.

Glial cells change their morphology and phenotype during normal aging to partially resemble reactive disease-associated microglial and astroglial cells (Boisvert et al., 2018; Krasemann et al., 2017; Spittau, 2017). Such alterations, coupled with age-induced neuroinflammation, create an environment which is permissive to synapse elimination and neuronal damage (Boisvert et al., 2018; von Bernhardi et al., 2015). Thus, vaccination with the AβCore-S construct reversed age-related microgliosis by upregulating homeostatic markers in microglia in both WT and Ts65Dn mice.

Reactive astrocytes, a characteristic of both AD and DS, exhibit higher levels of reactive oxygen species (ROS) and lower levels of synaptogenesis-related molecules (Chen et al., 2014). Conditionedmedia from DS-derived astrocytes confers toxicity to neurons and fails to promote neuronal ion channel maturation and synapse formation. Moreover, A1 astrocytes in DS lose the ability to promote neuronal growth, neuronal survival and synapse formation, failing to maintain tissue homeostasis (Liddelow et al., 2017). Also, reactive microglia induce A1-reactive astrocytes that secrete neurotoxins and complement components, promoting synapses degeneration (Liddelow et al., 2017). Levels of S100β are augmented in astrogliosis, and several reports have associated increased levels of S100β with the pathophysiology of degenerative and infectious/inflammatory brain disorders (Donato, 2001; Heizmann et al., 2002; Mrak and Griffin, 2001, 2004; Van Eldik and Wainwright, 2003). Since the human S100B gene maps to Hsa21 and is triplicated in DS (Chen et al., 2014), S100β is overexpressed in these patients. It has been found that S100β levels in severely affected brain regions of AD patients are higher than in age-matched control samples (Donato, 2001; Heizmann et al., 2002; Mrak and Griffin, 2001). Notably, the S100B gene is not triplicated in the Ts65Dn strain, but nevertheless exhibits higher expression in these mice possibly due to increased astrogliosis and disease-related reactivity. Astrocytes release S100β constitutively (Shashoua et al., 1984), and S100β release is augmented upon exposure of astrocytes to serotonin agonists (Whitaker-Azmitia et al., 1990), glutamate (Ciccarelli et al., 1999) or tumor necrosis factor-α (Edwards and Robinson, 2006). Once released, S100β can affect neurons, astrocytes, and microglia with different effects, depending on its concentration via engagement of the receptor for advanced glycation end products in large part (Donato, 2007).

We identified a reduction in the reactivity of S100β^+^ astrocytes in both the hippocampus and cortex of vaccinated Ts65Dn mice, consistent with reduced microglial activation in these mice. Both these observations provide evidence for the homeostatic effect of the AβCoreS vaccine.

In the adult normal brain, astrocytes exhibit a stellate morphology and show a slow rate of renewal (Farina et al., 2007; Skaper, 2007; Williams et al., 2007). However, in case of a brain insult, astrocytes rapidly retract their cytoplasmic processes, proliferate, and migrate to the site(s) of damage, giving rise to reactive gliosis (Farina et al., 2007; Skaper, 2007; Williams et al., 2007). These changes are largely dependent on alteration of the blood–brain barrier and are mediated by serum factors and locally released cytokines.

An increasing body of evidence suggests that S100β might have a role during neurogenesis, participating in astrocyte maturation (Raponi et al., 2007), and in migration of granule cell precursors (Hachem et al., 2007). Indeed, neurogenesis is severely compromised in DS from early developmental stage, featuring impaired neuronal precursor proliferation, slowing of cell cycle and altered differentiation (Rueda et al., 2012). Ts65Dn mice exhibit reduced neural precursor proliferation in the sub-ventricular zone (SVZ) (Ishihara et al., 2010; Rueda et al., 2012) and extended cell cycle in the CA3 at embryonic stages (Chakrabarti et al., 2007) and in the DG at early postnatal life (Contestabile et al., 2007). Additionally, cell proliferation is reduced in the SVZ of Ts65Dn mice from birth to adulthood (Bianchi et al., 2010a; Bianchi et al., 2010b; Trazzi et al., 2011). Finally, we found increased expression of complement 3 (C3) among astrocytes from sham-treated Ts65Dn mice. C3 is one of the most characteristic and highly upregulated genes in A1 astrocytes and is not expressed by ischemic A2 reactive astrocytes. Among human AD patients, 60% of GFAP-positive astrocytes are also positive for C3 (Liddelow et al., 2017), and therefore these cells probably play an integral role in disease initiation and progression. In MS, C3 astrocyte were found near demyelination lesions, and in close proximity with CD86-reactive microglia (Liddelow et al., 2017).

As a result of life-long overexpression of the APP and DYRK1a genes, DS individuals suffer from AD-related dementia in the vast majority of the cases over 40y. Therefore, targeting AD-related pathology, using the AβCore-S vaccine has the potential to slow the disease progression and the emergence of AD-related dementia. Indeed, age-dependent spatial-learning deficiency was alleviated in vaccinated Ts65Dn mice, as their performance in the Barnes maze imply a better hippocampal-dependent coding of the environment. Interestingly, we found age-related elevation in serum Aβ_1–40_ and Aβ_1–42_ among sham-treated Ts65Dn mice. This effect was moderated in vaccinated Ts65Dn mice. Thus, targeting Aβ throughout life resulted in reduced levels at 15 m of age. As the phenotype of microglial cells changes during life (Krasemann et al., 2017), we also found treatment-related effects of the AβCore-S vaccine on specific microglial markers and morphology. Levels of reactive markers, CD68 and Clec7a, were lower in vaccinated mice, and levels of homeostatic markers, 4D4 and P2RY12 were higher, compared with sham-treated mice. Additionally, reduced C3 expression by GFAP-positive astrocyte in the hippocampus of vaccinated Ts65Dn mice provides an evidence for therapeutic effect of the AβCore-S vaccine in reducing cellular reactivity and promoting restoration of homeostasis in the brain parenchyma.

## 5. Conclusions

In this study, we found that AD-related neuropathology and cognitive impairments found in the Ts65Dn mouse model of DS, can be ameliorated by a transient vaccination with the 1–11 fragment of murine Aβ. Since the origin and manifestation of AD-like neuropathology found in DS are largely different from sporadic and familial AD, especially in the onset of Aβ accumulation, we believe that DS individuals may benefit from active immunotherapy against Aβ from a young age.

## Supplementary Material

Supplementary Data
