# Supplementary material for: Restoring microglial and astroglial homeostasis using DNA immunization in a Down Syndrome mouse model
Source: Brain Behav Immun. Author manuscript; Available in PMC 2019 Feb 1. (PMC6358279; doi:10.1016/j.bbi.2018.10.004)
Supplement: Supplementary Data [file NIHMS1005590-supplement-Supplementary_Data.pdf]

Age (m)	Paradigm	Measurement	Figure(s)	Findings
3	Elevated zero maze	Baseline anxiety levels	S1B-E	Abnormal anxiety threshold in Ts65Dn mice
3	Open field	Baseline exploratory behavior	S1F-H	Motor hyper-activity in Ts65Dn mice
3	Radial arms water maze	Baseline long-term spatial learning, wet environment	S2	Ts65Dn mice exhibit a motor dysfunction in wet paradigms
3	Barnes maze	Baseline long-term spatial learning, dry environment	S3-4	Mild spatial learning deficit in Ts65Dn mice
6	Open field	Post-immunization exploratory behavior	S6B-D	Reduced motor hyper-activity post-immunization in Ts65Dn mice
6	Elevated zero maze	Post-immunization anxiety levels	S6E-H	Ts65Dm abnormal anxiety threshold remains unchanged post immunization
9	Barnes maze	Post-immunization long-term spatial learning	2B-H, S7	Reduced RM, WM errors post immunization; higher affinity to the target in vaccinated Ts65Dn mice
12	T-maze	Post-immunization short-term memory	3B	Short-term memory rescue post-immunization of Ts65Dn mice
12	Novel object recognition	Post-immunization short-term memory	3C	Short-term memory rescue post-immunization of Ts65Dn mice

Supplementary Table 1

Supplementary table 1. Behavioral and cognitive testing of vaccinated Ts65Dn mice. Ts65Dn mice and WT controls were tested prior to immunization to obtain a baseline measurement for anxiety (using the Elevated zero maze), exploratory behavior (using the Open field arena), and long-term spatial learning capacity (using the Radial arm water maze and the Barnes maze). Behavioral testing were conducted after immunization, at 6m of age and every 2-3 months until mice reached the age of 12m.

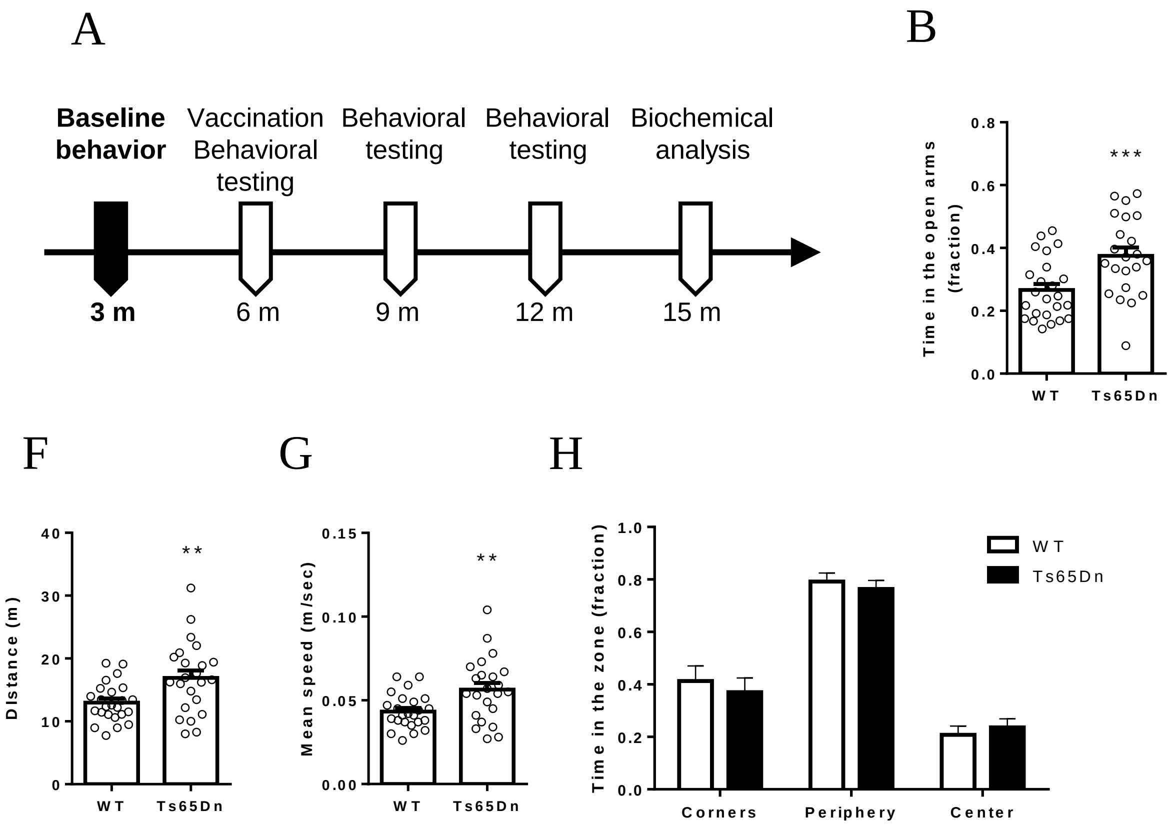

A
Baseline behavior
Vaccination Behavioral testing
Behavioral testing
Behavioral testing
Biochemical analysis
3 m
6 m
9 m
12 m
15 m
B
Time in the open arms (fraction)
***
WT
Ts65Dn
C
Distance (m)
WT
Ts65Dn
D
Mean speed (m/sec)
WT
Ts65Dn
E
Line crossing (count)
WT
Ts65Dn
F
Distance (m)
**
WT
Ts65Dn
G
Mean speed (m/sec)
**
WT
Ts65Dn
H
Time in the zone (fraction)
WT
Ts65Dn
Corners
Periphery
Center

Figure S1. Ts65Dn mice exhibit higher anxiety threshold and motor-hyperactivity in the Elevated zero maze and Open field test. (A) At 3m of age, Ts65Dn and WT mice were tested for anxiety in the EZM (B-E) and for exploratory behavior in the Open field test (F-H). **(B)** Ts65Dn mice spent a higher fraction of time in the open arms of the EZM than WT mice. **(C)** Total traveling distance, **(D)** mean speed and **(E)** number of line crossing, did not differ between strains in the EZM. **(F)** distance and **(G)** mean speed were higher in Ts65Dn mice compared with WT controls, **(H)** No strain effect was observed for time spent in the corners, periphery or center zones of the open field arena. Unpaired t-test, Two-way ANOVA ** $P<0.01$, *** $P<0.001$.

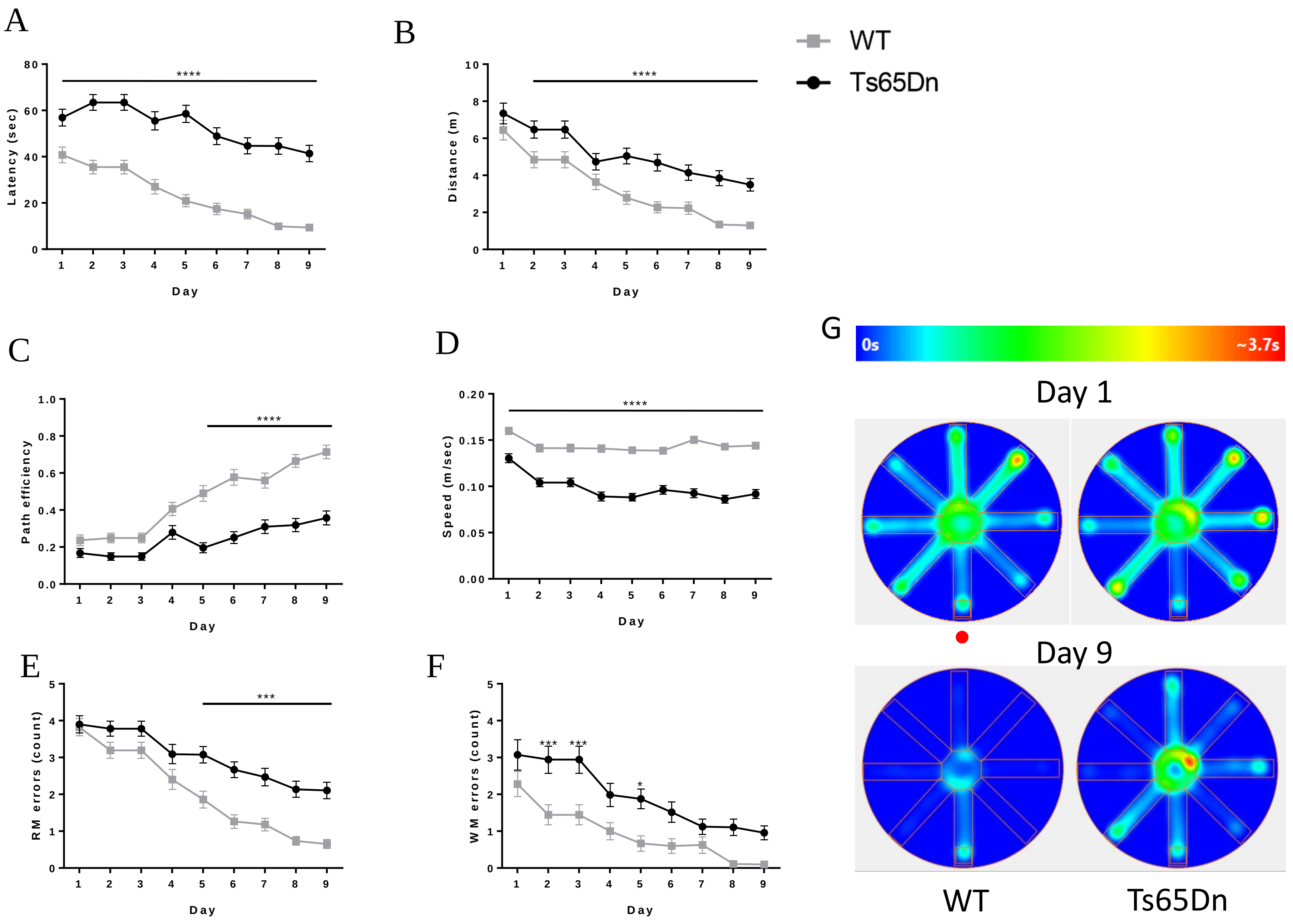

A
B
WT
Ts65Dn
Latency (sec)
Distance (m)
Day
****
C
D
Path efficiency
Speed (m/sec)
E
F
RM errors (count)
WM errors (count)
***
*
G
0s
~3.7s
Day 1
Day 9
WT
Ts65Dn

Figure S2. Cognitive and motor deficits of Ts65Dn mice in the Radial arm water maze. Ts65Dn and WT mice were tested for spatial learning capacity using the RAWM at 3m of age. Ts65Dn mice exhibit **(A)** higher latency to reach the platform throughout the acquisition phase ($P<0.0001$), **(B)** elevated travelling in days 2-9 of acquisition ($P<0.0001$). **(C)** Reduced path efficiency of Ts65Dn mice is reduced in days 5-9 of acquisition ($P<0.0001$), and **(D)** lower swimming speed ($P<0.0001$), compared with WT mice. **(E)** Reference memory error rate was higher in Ts65Dn mice in days 5-9 of acquisition ($P<0.001$) and **(F)** higher working memory error rate in days 2,3,5, compared with WT mice (P<0.001, 0.001,0.05 respectively), compared with WT controls **(G)** Heat maps of mice center-point location, at day 1 (WT: left upper panel, Ts65Dn: right upper panel) and 9 (bottom panels, accordingly), indicating Ts65Dn but not WT mice had visited non-target arms at the last day of acquisition. Repeated-measures Two-way ANOVA, *** $P<0.001$, **** $P<0.0001$.

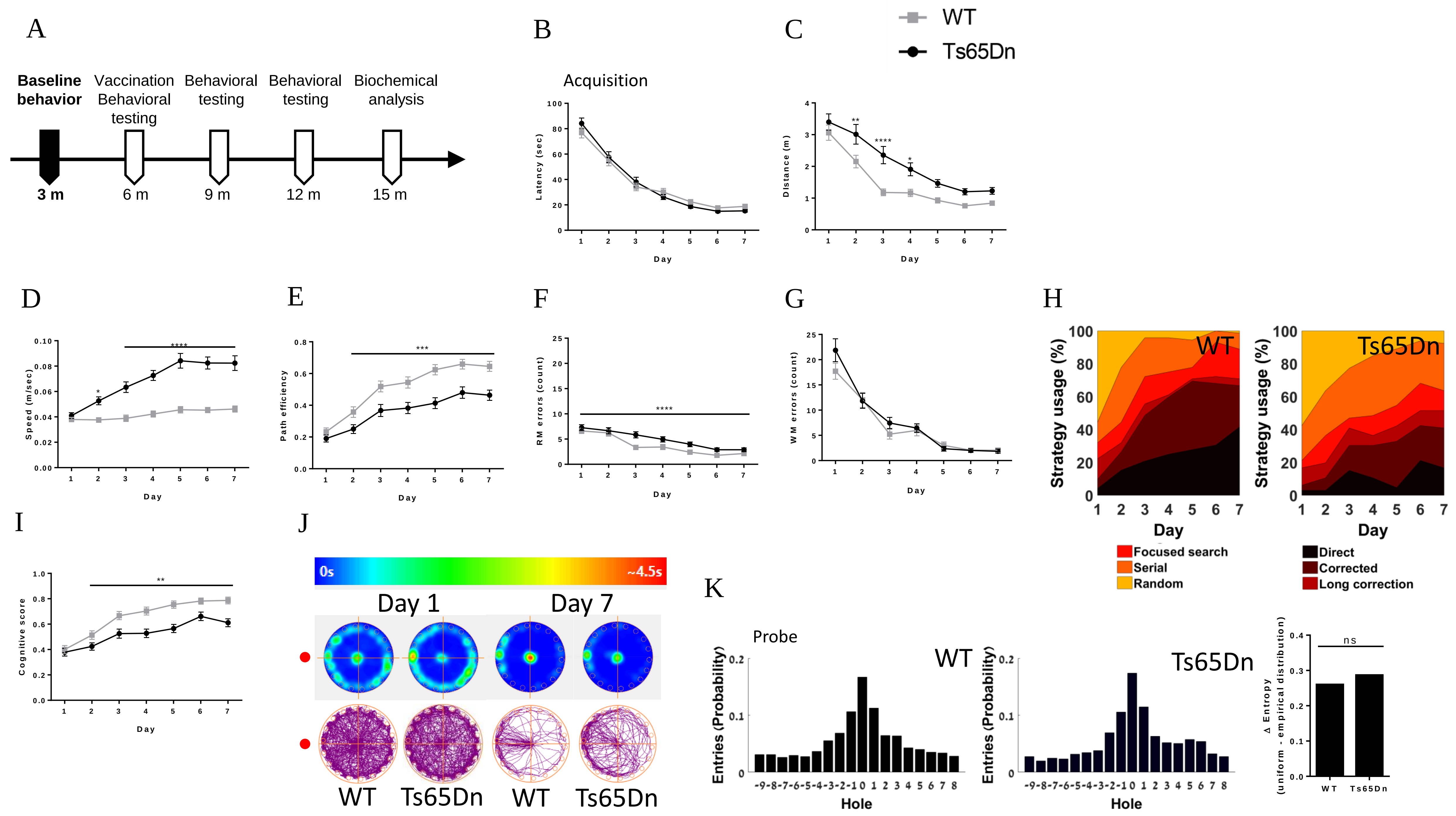

A
Baseline behavior
Vaccination Behavioral testing
Behavioral testing
Behavioral testing
Biochemical analysis
3 m
6 m
9 m
12 m
15 m
B
Acquisition
Latency (sec)
Day
C
WT
Ts65Dn
Distance (m)
Day
D
Speed (m/sec)
Day
E
Path efficiency
Day
F
RM errors (count)
Day
G
WM errors (count)
Day
H
WT
Ts65Dn
Strategy usage (%)
Day
Focused search
Serial
Random
Direct
Corrected
Long correction
I
Cognitive score
Day
J
0s
~4.5s
Day 1
Day 7
WT
Ts65Dn
WT
Ts65Dn
K
Probe
WT
Ts65Dn
Entries (Probability)
Hole
Δ Entropy (uniform - empirical distribution)
ns

Figure S3. Ts65Dn mice exhibit lower spatial learning capacity at baseline measurement. (A) Prior to immunization, 3m-old Ts65Dn and WT controls were tested for baseline cognitive capacity using the Barnes maze. In the acquisition phase, **(B)** latency to reach the target was similar in DS and WT (P=0.98), **(C)** Ts65Dn mice travelled a longer distance than WT mice in days 2-4 (P<0.01, P<0.0001, P<0.05, respectively), **(D)** Ts65Dn mice travelled at a higher speed than WT mice in days 2-7 (P<0.05, P<0.0001) **(E)** Ts65Dn mice exhibit lower path efficiency than WT mice (P<0.001)**,** and **(F)** elevated RM rate (P<0.0001) but not **(G)** WM rate (P=0.16) errors. **(H)** Spatial strategy analysis and **(I)** quantification into cognitive scores reveals higher learning ability in the WT strain (P<0.01). **(J)** Heat maps and trajectory plots in the first and last days of the acquisition phase of Barnes maze. In the probe test **(K)** no between-strain difference was observed for the distribution of hole-entries (left and middle panels), resulted in similar Δ entropy (uniform – empirical distributions, right panel). Repeated-measures two-way ANOVA, Two-sample Kolmogorov-Smirnov test. ** P<0.01, *** P<0.001, **** P<0.0001.

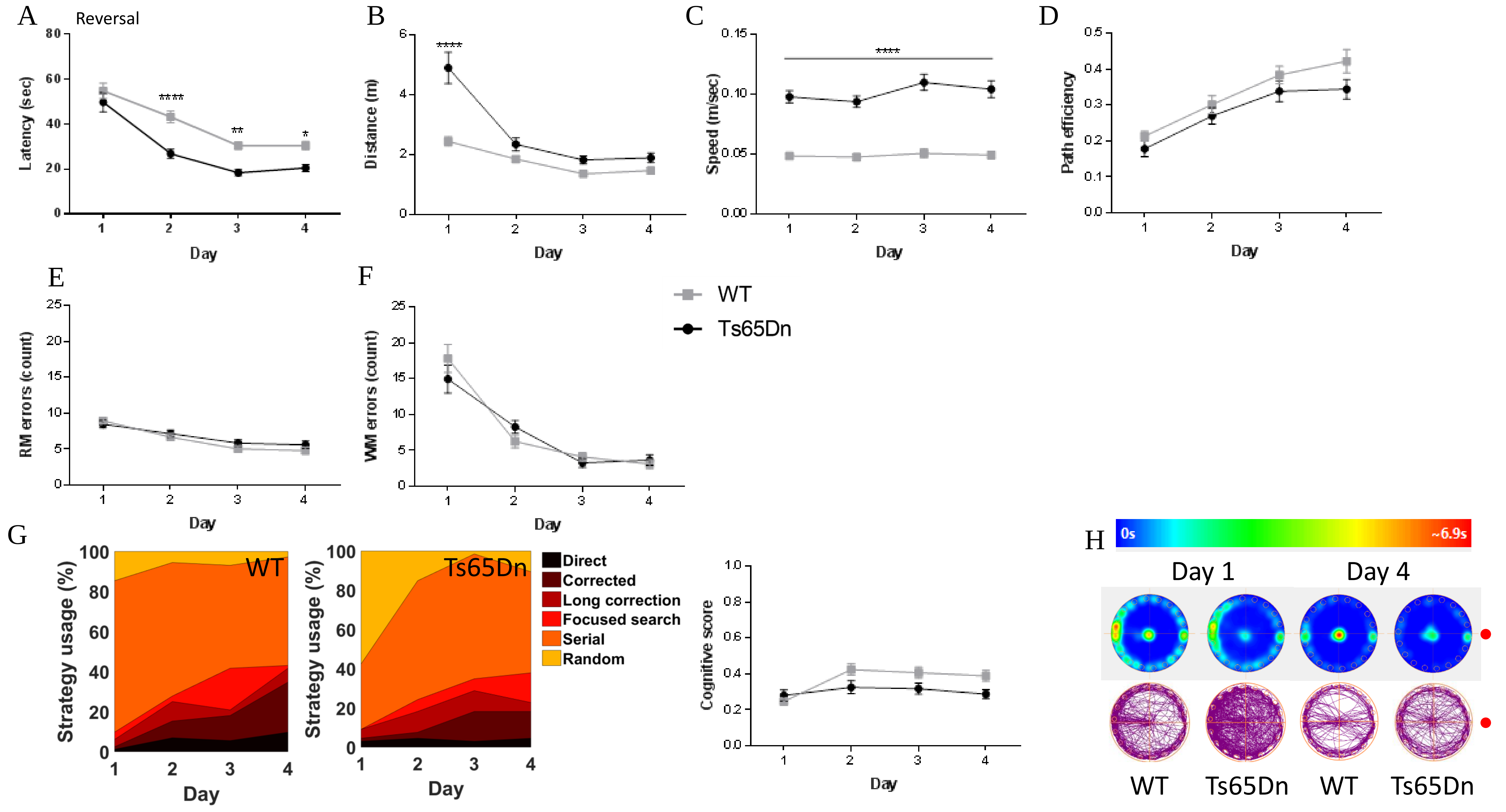

A
Reversal
Latency (sec)
Day
B
Distance (m)
C
Speed (m/sec)
D
Path efficiency
E
RM errors (count)
F
WM errors (count)
WT
Ts65Dn
G
Strategy usage (%)
Direct
Corrected
Long correction
Focused search
Serial
Random
Cognitive score
H
0s
~6.9s
Day 1
Day 4

Figure S4. Ts65Dn mice exhibit lowered spatial memory retention in the reversal task of the Barnes maze. Following the acquisition phase, mice were tested in the reversal task. **(A)** Latency to reach the target was lower in Ts65Dn mice compared with WT controls in days 2-4 (P<0.0001,0.01,0.05, respectively). **(B)** Swimming distance was higher in Ts65Dn mice in day 1 (P<0.0001). **(C)** Ts65Dn mice exhibit elevated swimming speed throughout the reversal task, (P<0.0001). **(D)** Path efficiency, **(E)** reference and **(F)** working memory error rate did not differ between strains. **(G)** Spatial strategy analysis reveals that although WT mice used the *direct* are *corrected* strategy at a higher rate than Ts65Dn by the last day (34, 18%, respectively), the most prevalent strategy among both groups was *serial search* (54, 51%, respectively). Accordingly, cognitive scores did not differ between groups. **(H)** Heat maps (upper panels) and trajectory plot (bottom panels) of the first and last days of the reversal task reveals memory retention in WT mice, as this group but not the Ts65Dn group visit the old target profoundly by the last day. Repeated-measures Two-way ANOVA, * P<0.05, ** P<0.01, **** P<0.0001.

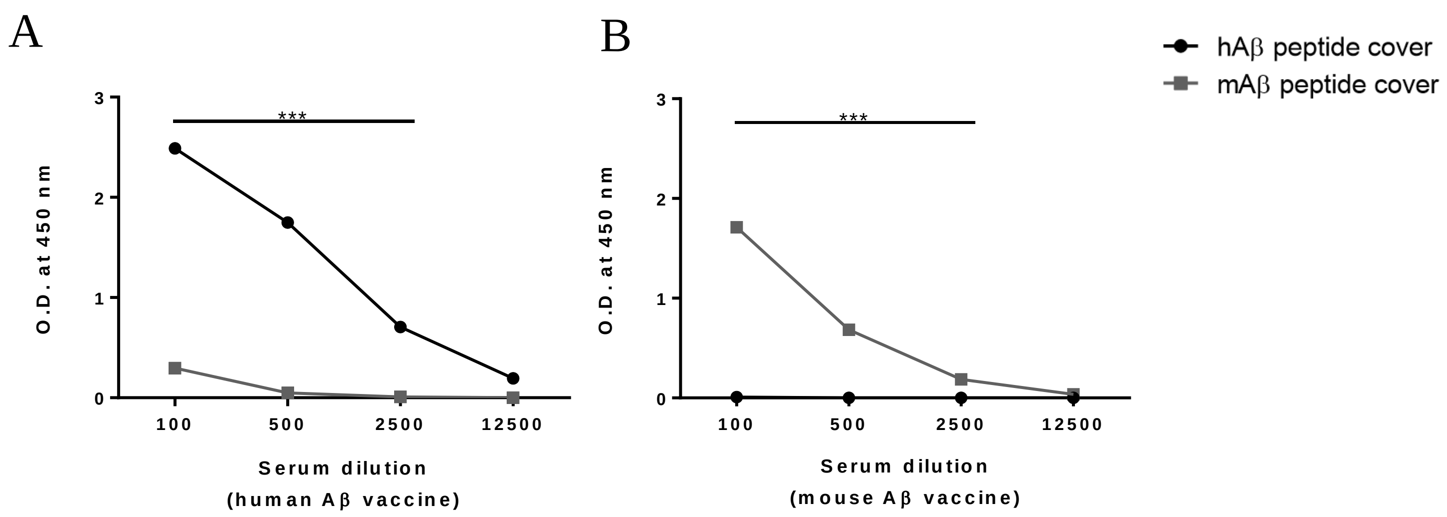
A
B
hAβ peptide cover
mAβ peptide cover
O.D. at 450 nm
3
2
1
0
***
100
500
2500
12500
Serum dilution
(human Aβ vaccine)
O.D. at 450 nm
3
2
1
0
***
100
500
2500
12500
Serum dilution
(mouse Aβ vaccine)

Figure S5. Vaccine-derived anti-human and anti-murine Aβ antibodies show low cross reactivity. Affinity and cross reactivity of anti-human and murine Aβ were tested using ELISA. Briefly, ELISA plates were coated with either human or murine recombinant Aβ. Affinity was tested by applying serum from mice vaccinated against the appropriate specie of Aβ and cross reactivity was tested by applying the opposite. **(A)** Antibodies found in the serum mice vaccinated against human Aβ, detect human but not murine Aβ, $P<0.001$. **(B)** Antibodies found in the serum mice vaccinated against murine Aβ, detect murine but not human Aβ, $P<0.001$. Repeated-measures Two-way ANOVA, *** $P<0.001$.

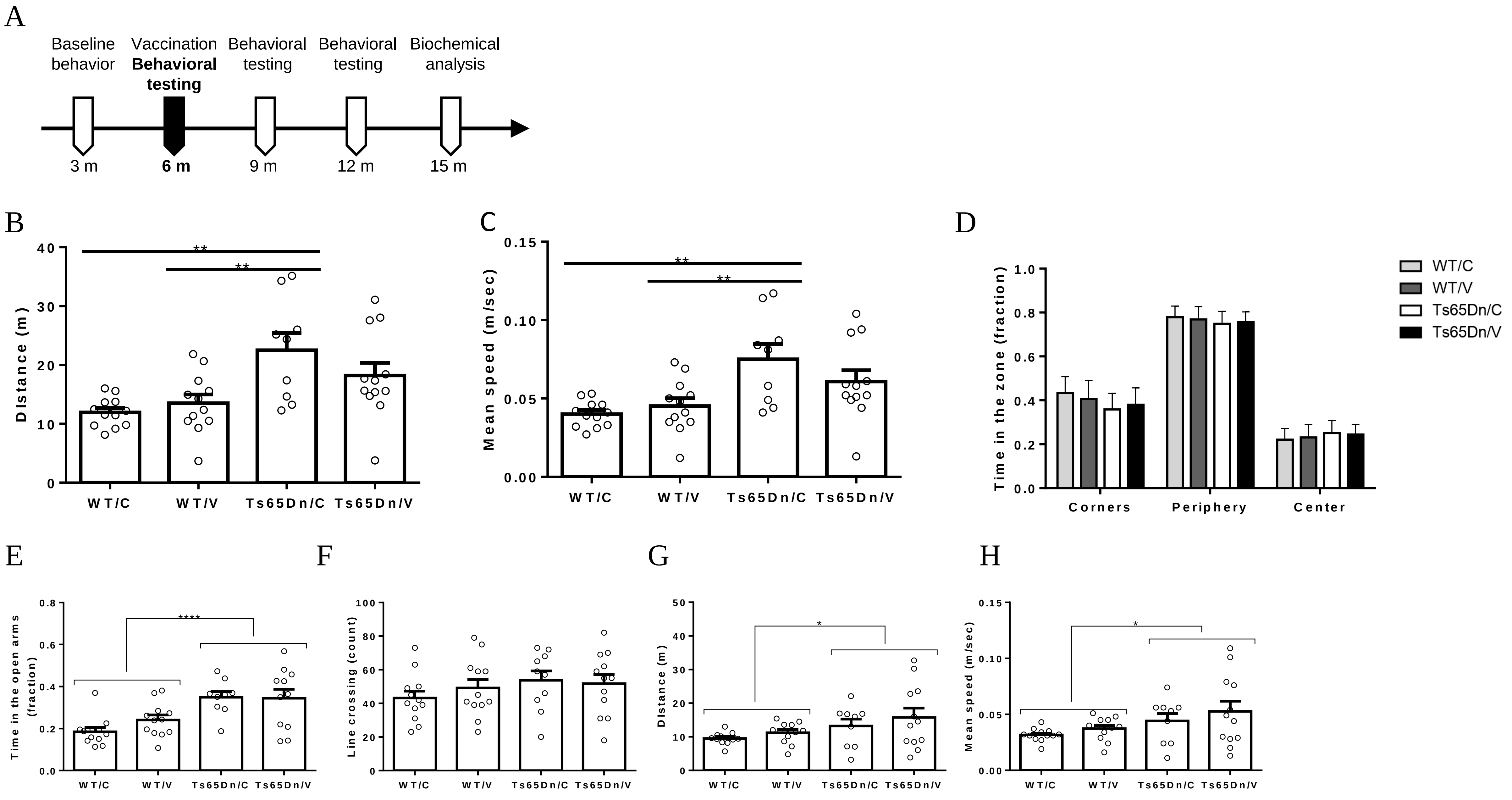

A
Baseline behavior
Vaccination Behavioral testing
Behavioral testing
Behavioral testing
Biochemical analysis
3 m
6 m
9 m
12 m
15 m
B
Distance (m)
WT/C
WT/V
Ts65Dn/C
Ts65Dn/V
C
Mean speed (m/sec)
D
Time in the zone (fraction)
Corners
Periphery
Center
WT/C
WT/V
Ts65Dn/C
Ts65Dn/V
E
Time in the open arms (fraction)
F
Line crossing (count)
G
Distance (m)
H
Mean speed (m/sec)

Figure S6. Vaccinating Ts65Dn mice with AβCore-S reduces motor-hyperactivity and does not affect anxiety phenotype. (A) Post immunization, Ts65Dn and WT mice were tested for exploratory behavior using the Open field test (B-D) and anxiety using the Elevated zero maze (E-H). **(B)** sham-vaccinated Ts65Dn mice travelled a longer distance in the OF arena compared with WT mice, $P<0.01$, whereas vaccinated Ts65Dn mice resembled WT controls. **(C)** Accordingly, the speed of sham-vaccinated Ts65Dn mice was higher compared with WT mice, $P<0.01$. **(D)** Time spent in the corners, periphery and center zones of the OF arena did not differ between groups. **(E)** Both vaccinated and sham-vaccinated Ts65Dn mice spent a higher fraction of time in the open zones of the EZM compared with WT controls, $P<0.0001$. **(F)** Number of line crossing did not differ between groups. **(G)** Distance and **(H)** speed of movement in the EZM was higher among both vaccinated and sham-vaccinated Ts65Dn mice compared with WT controls, $P<0.05$. One-way and Two-way ANOVA, * $P<0.05$, ** $P<0.01$, **** $P<0.0001$.

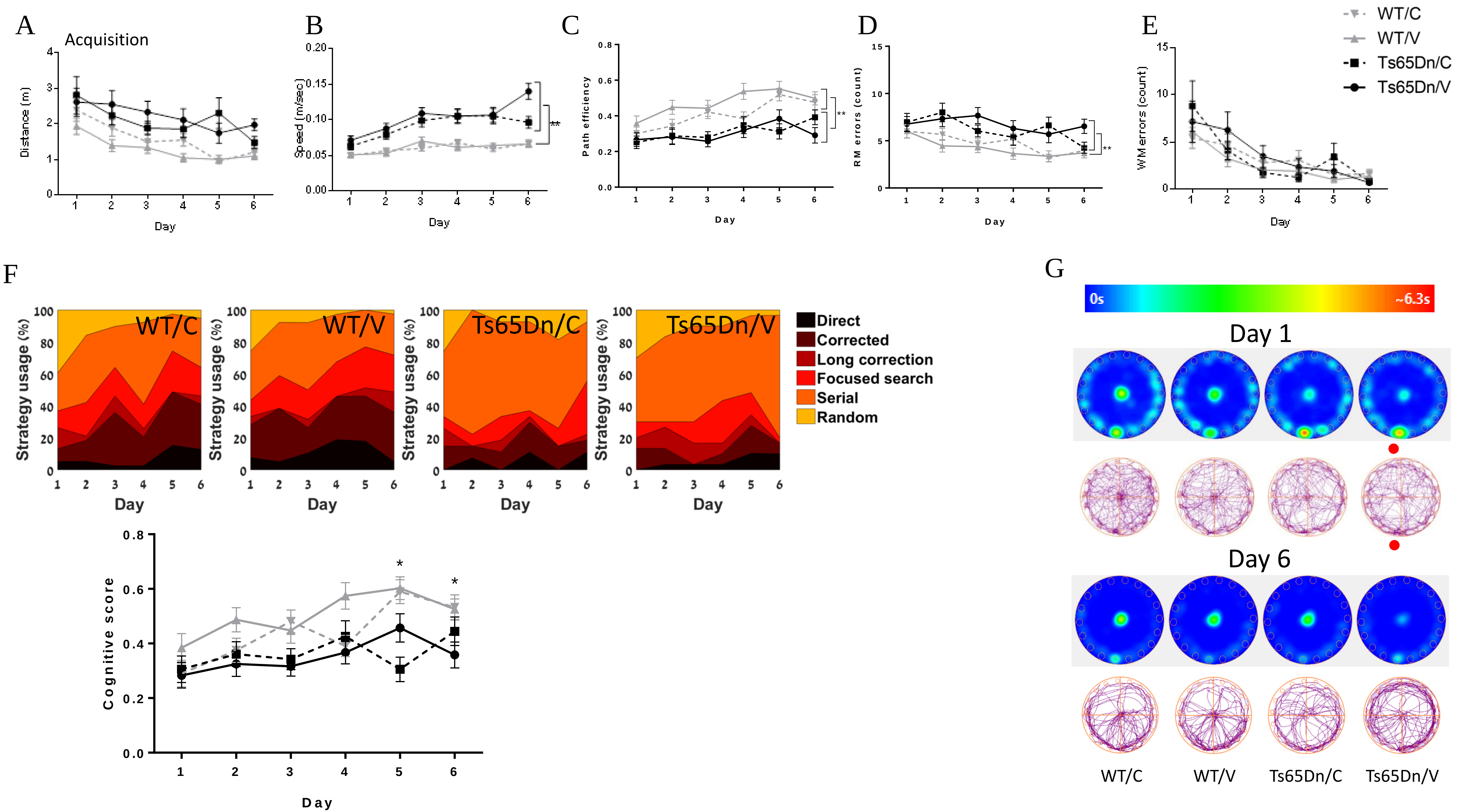

A
Acquisition
Distance (m)
Day
B
Speed (m/sec)
Day
**
C
Path efficiency
Day
**
D
RM errors (count)
Day
**
E
WM errors (count)
Day
WT/C
WT/V
Ts65Dn/C
Ts65Dn/V
F
Strategy usage (%)
WT/C
WT/V
Ts65Dn/C
Ts65Dn/V
Day
Direct
Corrected
Long correction
Focused search
Serial
Random
Cognitive score
Day
*
*
G
0s
~6.3s
Day 1
Day 6
WT/C
WT/V
Ts65Dn/C
Ts65Dn/V

Figure S7. Vaccinated Ts65Dn mice obtain better results in the probe test but not in the acquisitions phase of Barnes maze at 9m of age. At 9m of age, mice were tested for cognitive capacity using the Barnes maze and probe test (main figure 2). In the acquisition phase **(A)** total distance travelled did not differ between groups. However, vaccinated and sham-vaccinated Ts65Dn mice exhibit **(B)** higher travelling speed, **(C)** lowered path efficiency and **(D)** higher reference memory error rate, compared with WT controls, P<0.01. **(E)** Working memory errors did not differ between strains. **(F)** Strategy analysis reveals that Ts65Dn mice utilized *direct* and *corrected* at a lower rate than WT mice by the last day of acquisition (17m 38.5%, respectively), reflected in lower cognitive scores in days 5-6 of acquisition (lower panel), P<0.05. **(G)** Heat maps (top panels) and trajectory plots (bottom panels) with no significant treatment effects. Repeated-measures Two-way ANOVA, *P<0.05, ** P<0.01.
